# AI-Driven Enzyme Engineering: Emerging Models and Next-Generation Biotechnological Applications

**DOI:** 10.3390/molecules31010045

**Published:** 2025-12-22

**Authors:** Mohd Faheem Khan, Mohd Tasleem Khan

**Affiliations:** 1 UCD School of Agriculture and Food Science, University College Dublin, Belfield, Dublin 4, D04 V1W8 Dublin, Ireland; 2 School of Engineering & Physical Sciences, Heriot-Watt University, Edinburgh Campus, Edinburgh EH14 4AS, UK

**Keywords:** enzyme engineering, artificial intelligence, machine learning, protein structure, biocatalysis, mutagenesis, applications

## Abstract

Enzyme engineering drives innovation in biotechnology, medicine, and industry, yet conventional approaches remain limited by labour-intensive workflows, high costs, and narrow sequence diversity. Artificial intelligence (AI) is revolutionising this field by enabling rapid, precise, and data-driven enzyme design. Machine learning and deep learning models such as AlphaFold2, RoseTTAFold, ProGen, and ESM-2 accurately predict enzyme structure, stability, and catalytic function, facilitating rational mutagenesis and optimisation. Generative models, including ProteinGAN and variational autoencoders, enable de novo sequence creation with customised activity, while reinforcement learning enhances mutation selection and functional prediction. Hybrid AI–experimental workflows combine predictive modelling with high-throughput screening, accelerating discovery and reducing experimental demand. These strategies have led to the development of synthetic “synzymes” capable of catalysing non-natural reactions, broadening applications in pharmaceuticals, biofuels, and environmental remediation. The integration of AI-based retrosynthesis and pathway modelling further advances metabolic and process optimisation. Together, these innovations signify a shift from empirical, trial-and-error methods to predictive, computationally guided design. The novelty of this work lies in presenting a unified synthesis of emerging AI methodologies that collectively define the next generation of enzyme engineering, enabling the creation of sustainable, efficient, and functionally versatile biocatalysts.

## 1. Introduction

Enzymes are essential biological catalysts in biotechnology, pharmaceuticals, and industrial chemistry, owing to their exceptional catalytic efficiency, high substrate specificity, and environmentally sustainable characteristics. They facilitate diverse bioprocesses, including biofuel and food production, drug synthesis, diagnostics, and therapeutic interventions, while promoting cleaner, more cost-effective manufacturing through reduced hazardous chemical usage and minimised waste generation [1]. However, despite their immense potential, the rational design and optimisation of enzymes remain a formidable challenge. Conventional enzyme engineering approaches, such as directed evolution and rational design, rely heavily on iterative cycles of random mutagenesis and high-throughput screening, which are labour-intensive, time-consuming, and costly [2]. These approaches often explore only a narrow region of the protein fitness landscape, limiting access to novel or improved enzyme functions beyond known sequence scaffolds.

Progress in enzyme engineering is hindered by several challenges, including the complex and non-linear sequence–structure–function relationship, the scarcity of kinetic, thermodynamic, and structural data, and the difficulty of predicting synergistic mutation effects [3,4]. Moreover, the vast combinatorial sequence space makes exhaustive mutagenesis and screening impractical. Consequently, new strategies are needed to efficiently explore this landscape [2,3]. Artificial intelligence and machine learning offer powerful solutions by mining large-scale biological datasets to reveal patterns, predict functional outcomes, and guide experiments [3]. Unlike traditional empirical approaches, AI can capture non-linear interactions among residues, cofactors, and substrates, enabling the modelling of complex enzyme behaviours that are otherwise difficult to predict using conventional methods [2,3,4].

**Figure 1:**
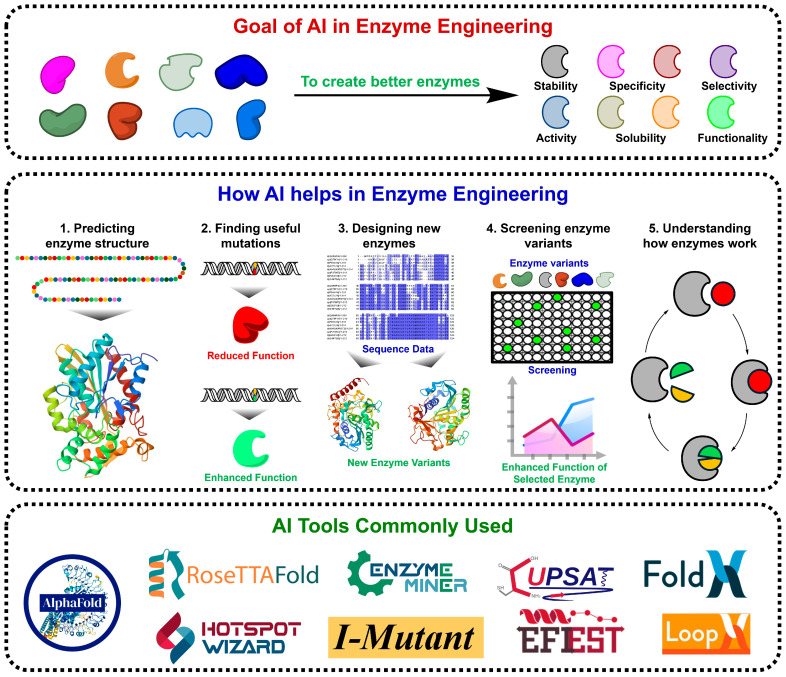
**Overview of AI in Enzyme Engineering.** This figure summarises the role of AI in developing enzymes with enhanced activity, stability, specificity, and overall functionality. AI supports structure prediction, mutation identification, variant design, screening, and mechanistic analysis, collectively accelerating the discovery and optimisation of next-generation biocatalysts.

Recent advances in AI-guided enzyme engineering have transformed the field by integrating machine learning (ML), deep learning (DL), and generative models, enabling rapid, data-driven prediction and optimisation of enzyme properties such as activity, stability, specificity, and solubility [4]. AI models not only learn from experimental and computational datasets but also continuously improve as more data become available, allowing them to propose rational mutations and predict beneficial variants with unprecedented accuracy [5]. These approaches bridge computational modelling with experimental validation, significantly reducing both time and cost compared with traditional workflows. Figure 1 illustrates the AI-driven enzyme engineering framework, outlining its objectives, workflow, and tools that enhance enzymatic features through a comprehensive five-step process comprising structure prediction, mutation identification, variant design, functional screening, and mechanistic interpretation. Widely adopted platforms, including AlphaFold, RoseTTAFold, EnzymeMiner, HotSpot Wizard, and I-Mutant, exemplify the practical application of AI for accelerated enzyme discovery and optimisation.

AI-powered autonomous design platforms that integrate large language models with biofoundry automation have demonstrated remarkable catalytic improvements, achieving up to 90-fold increases in substrate specificity within weeks [5]. Similarly, ML-guided cell-free expression systems can assess over 10,000 reactions, successfully identifying enzyme variants with up to 42-fold enhanced catalytic efficiency [6]. These integrated AI-experimental workflows combine predictive modelling with high-throughput experimentation, enabling the rapid and targeted discovery of high-performance biocatalysts for a wide range of chemical transformations. Complementary computational tools, including molecular dynamics (MD) simulations, Rosetta-based energy scoring, and advanced structure prediction models, are reshaping structure-guided enzyme engineering by providing insights into conformational dynamics critical for catalysis and improving the precision of activity predictions [7,8].

Beyond optimising natural enzymes, AI has facilitated the development of synthetic enzymes, or “synzymes”, which replicate or surpass natural catalytic functions while maintaining enhanced stability and adaptability under extreme physicochemical conditions [9]. The convergence of AI-driven molecular design, high-throughput screening, and AI-based pathway optimisation has also advanced biocatalytic route engineering in synthetic biology. Retrosynthetic algorithms and enzyme function predictors now propose feasible pathways for complex molecule synthesis and sustainable chemical production [10]. As Ferreira et al. (2022) emphasise, these advances mark a shift from empirical, trial-and-error approaches to predictive, data-guided enzyme design, establishing a rational and scalable framework for next-generation biocatalyst discovery [11].

The integration of AI into enzyme engineering is not merely a technological trend but a scientific necessity. It offers a means to overcome long-standing barriers imposed by limited data, complex mutational interactions, and experimental constraints, ultimately enabling a rational, predictive, and accelerated pathway to enzyme innovation. Collectively, AI-enabled enzyme engineering represents a paradigm shift that integrates computational foresight with experimental validation, setting the stage for transformative applications across biotechnology, medicine, and environmental science.

## 2. Core AI Techniques in Enzyme Engineering

**Figure 2:**
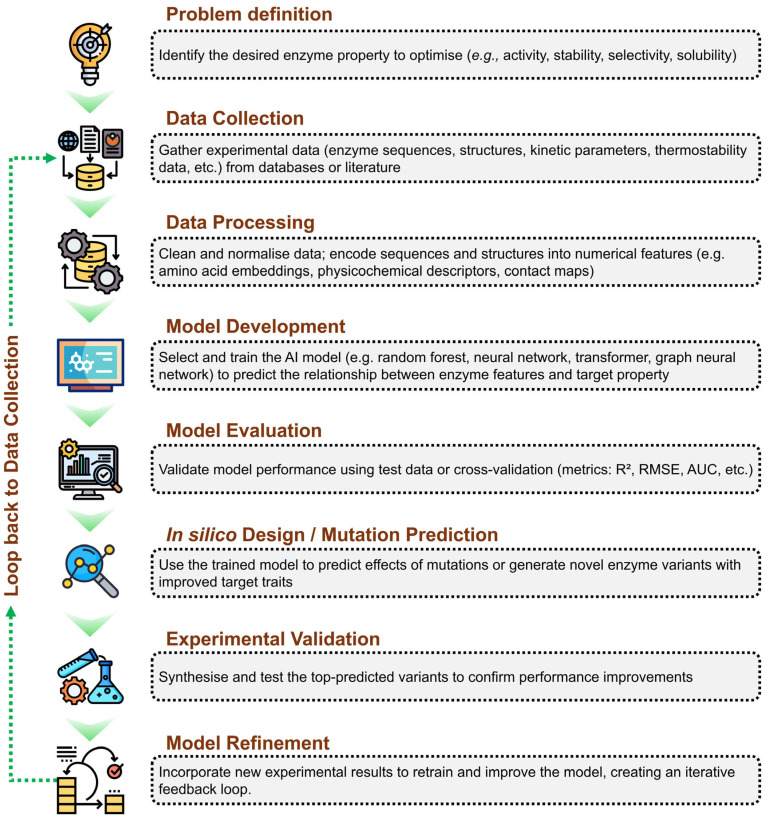
**Workflow of an AI-driven enzyme engineering approach.** Stepwise representation of the artificial intelligence-based workflow for enzyme optimisation, including problem definition, data collection and processing, model development and evaluation, in silico mutation prediction, experimental validation, and iterative model refinement through feedback integration.

The artificial intelligence-driven enzyme engineering workflow begins with clearly defining the target property to optimise, such as activity or stability. Experimental data, including sequences, structures, and kinetic parameters, are collected and pre-processed into suitable numerical representations. Machine learning models are then developed and trained to learn the relationship between enzyme features and desired properties, followed by rigorous performance evaluation. The trained model is subsequently used to predict beneficial mutations or design improved variants in silico. Top candidates are experimentally validated, and the resulting data are integrated back into the model, establishing an iterative feedback loop that continuously enhances predictive accuracy and enzyme performance. Figure 2 summarises this workflow, highlighting the sequential steps from problem definition to model refinement and illustrating the iterative loop where experimental data feed back into the AI system to enhance future predictions. This framework exemplifies how AI facilitates efficient, data-driven design cycles, accelerating enzyme discovery and optimisation beyond traditional trial-and-error methods.

Table 1 provides representative examples of widely used AI tools for enzyme and protein engineering, summarising their functions, key features, applications, developers, and source links.

Recent advances in AI have revolutionised enzyme engineering by enabling precise prediction, design, and optimisation of biocatalysts at an unprecedented scale. Core AI techniques such as ML and deep learning (DL) are now integral to predicting enzyme thermostability, catalytic efficiency, and substrate specificity using diverse algorithms, including Random Forests, Support Vector Machines (SVM), Gradient Boosting, and advanced neural architectures like CNNs, RNNs, and Transformers (e.g., AlphaFold, ESMFold) [12]. Generative models, such as Variational Autoencoders (VAEs) and Generative Adversarial Networks (GANs), extend these capabilities by creating novel enzyme variants with tailored properties, while protein language models (e.g., ProGen, ESM-2) leverage massive sequence datasets to infer functional sequence–structure relationships [12,13]. Reinforcement learning (RL) further enhances the design process by dynamically optimising mutagenesis strategies, balancing the exploration of new sequences with the exploitation of known beneficial mutations [14]. Looking ahead, quantum computing holds promise for simulating enzyme catalysis with molecular-level precision, potentially enabling the accurate modelling of reaction pathways and catalytic mechanisms beyond classical computational limits. These integrated AI systems have established a robust computational infrastructure that accelerates enzyme discovery, reduces experimental costs, and drives innovation in next-generation biocatalyst development.

### 2.1. Machine Learning (ML) Models

Enzyme engineering seeks to enhance catalytic activity, stability, and substrate specificity for applications across biotechnology, medicine, and sustainable chemistry [4,15]. Traditional approaches, such as rational design and directed evolution, have achieved significant success but remain limited by the vastness of protein sequence space and the labour-intensive nature of experimental screening. In recent years, ML has emerged as a powerful tool for predicting, optimising, and designing enzymes with unprecedented efficiency [2].

#### 2.1.1. Predictive Models and Data-Driven Design

ML algorithms, including Random Forests, Support Vector Machines (SVM), Gradient Boosting, and ridge regression, can predict enzyme thermostability, catalytic efficiency, and substrate specificity by identifying complex sequence–function relationships [4,16]. For example, Landwehr et al. demonstrated a cell-free, ML-guided platform integrating DNA assembly and functional assays to engineer amide synthetases [6]. Their models predicted enzyme variants with 1.6- to 42-fold higher activity compared to the parental enzymes, showing the strong predictive power of ML in biocatalyst optimisation. Similarly, Thomas et al. developed TeleProt, an ML framework that merges evolutionary and assay data to design diverse protein libraries. TeleProt outperformed traditional directed evolution by discovering a nuclease enzyme with an 11-fold increase in catalytic activity [17]. Liu et al. also used ML-guided protein engineering to enhance transaminase performance under neutral pH, achieving a 3.7-fold improvement [18]. These examples highlight how data-driven modelling accelerates enzyme discovery and functional optimisation while minimising experimental effort.

#### 2.1.2. ML for Enzyme Function and Active Site Prediction

ML has transformed enzyme engineering by enabling accurate prediction of enzyme function and catalytic residues, surpassing the limitations of homology-based bioinformatics. Traditional tools such as BLAST+ (v2.17.0) [19], PROSITE (v2025_01) [20], and Pfam (v38.0) [21] rely on sequence similarity to infer enzyme function, yet they perform poorly for distantly related or novel proteins. ML approaches overcome these constraints by learning nonlinear relationships from diverse sequence and structural descriptors, allowing robust functional predictions even in the absence of close homologs.

The Enzyme Commission (EC) system provides a hierarchical framework for categorising enzymatic reactions, and ML models can now infer EC numbers directly from raw sequences. Early models using Support Vector Machines or Random Forests [22,23] required handcrafted features, whereas recent deep learning frameworks such as DEEPre (v1.0) [24], ECPred (v1.1) [25], mlDEEPre (v1.0) [26], and DeepEC (v1.0) [27] automatically extract representations, achieving improved generalisation and accuracy across enzyme classes.

For catalytic site identification, structure-based ML models such as PREvaIL [28] and 3D convolutional neural networks [29] analyse spatial and physicochemical features, attaining precision above 90%. Collectively, these advances integrate sequence- and structure-level learning to accelerate enzyme function discovery and mechanism elucidation.

#### 2.1.3. Emerging Trends in Data-Driven Enzyme Design

Advances in deep learning and generative models further expand the possibilities for enzyme design. Protein language models, structural graph networks, and zero-shot learning approaches can generate novel enzyme sequences and predict functionality directly from amino acid data [30]. Such models can explore unexplored regions of the protein fitness landscape and suggest beneficial mutations without exhaustive experimentation.

Despite these advances, challenges persist in ensuring data quality, model interpretability, and generalisation. The adoption of standardised datasets, robust validation protocols, and transparent reporting practices remains essential for building reliable, reproducible ML frameworks [31]. Machine learning is transforming enzyme engineering from trial-and-error experimentation into a predictive, data-driven science. By coupling high-throughput experimentation with intelligent algorithms, researchers can now explore vast protein sequence spaces efficiently, leading to faster development of stable, selective, and sustainable biocatalysts for industrial and biomedical applications.

### 2.2. Deep Learning (DL) Models

Deep learning (DL) has revolutionised protein science by enabling end-to-end prediction and design using amino acid sequences and three-dimensional (3D) structural information. Architectures such as Convolutional Neural Networks (CNNs), Recurrent Neural Networks (RNNs), and Transformer-based models (e.g., AlphaFold and ESMFold) have demonstrated unprecedented accuracy in predicting protein folds and dynamics [32,33]. These innovations have redefined our ability to interpret the complex sequence–structure–function relationships that underpin biological activity, catalysis, and regulation. Transformer-based models leverage attention mechanisms to capture both local and long-range dependencies across sequences, enabling highly precise structure prediction and functional annotation. AlphaFold, for instance, predicts atomic-level 3D structures from sequence alone, while ESMFold extends this capability with large-scale protein language modelling, facilitating rapid analysis of massive sequence datasets [34,35]. In particular, RNNs and their variant, long short-term memory (LSTM) networks, excel at modelling sequential dependencies, capturing long-range relationships within protein sequences [36,37,38,39].

#### 2.2.1. Designing Dynamic and Functional Proteins

Traditional protein design primarily focuses on static, single-state structures. However, biological proteins often rely on dynamic conformational changes for their function, such as enzyme catalysis and signal transduction. Guo et al. addressed this challenge by developing a deep learning–guided framework to design dynamic proteins capable of allosteric regulation [40]. Their method integrated AlphaFold2 predictions with molecular dynamics simulations to identify sequence variants that can populate multiple conformational states. Experimental validation confirmed the successful design of proteins whose equilibrium between states could be tuned by ligand binding or distal mutations. This demonstrated that deep learning models not only predict static structures but also help engineer proteins with programmable conformational flexibility is a crucial step toward synthetic signal-responsive systems.

#### 2.2.2. Predicting and Optimising Enzyme Function

Beyond structure prediction, DL has transformed enzyme engineering by enabling data-driven discovery of mutants with enhanced activity, stability, or specificity. Jiang et al. introduced PRIME, a temperature-aware language model that predicts protein mutants with improved thermal stability and catalytic activity without prior mutagenesis data [41]. Similarly, Wang et al. developed CataPro, a model combining pretrained embeddings and molecular fingerprints to accurately predict kinetic parameters such as turnover number (*k_cat_*) and catalytic efficiency (*k_cat_*/*K_m_*) [42]. These approaches outperform traditional physics-based simulations, offering scalable and generalizable tools for enzyme optimisation.

Complementary models such as DEKP [43] and EnzyACT [44] employ graph neural networks and multimodal embeddings to represent protein sequence–structure interactions. They predict the effects of single or multiple mutations on enzyme activity, addressing the long-standing trade-off between stability and activity. Moreover, zero-shot predictors, DL systems that infer mutation effects without labelled data, are opening new avenues for rapid enzyme optimisation [45].

#### 2.2.3. Toward Intelligent, Generalizable Protein Design

The convergence of protein language models, generative diffusion models, and multimodal architectures has ushered in a new paradigm of function-driven structural innovation [46]. Rather than merely analysing structure to infer function, these models autonomously generate proteins that meet desired catalytic or regulatory criteria, effectively breaking the constraints of natural evolution. Integrating DL with physics-based and experimental validation frameworks allows the rational design of enzymes that perform non-natural reactions, expand biocatalytic toolboxes, and enable sustainable biomanufacturing [47].

Deep learning has transformed protein science from descriptive to predictive and generative. Through architectures like CNNs, RNNs, and Transformers, DL models can capture the intricate sequence–structure–dynamics relationships governing biological function. From AlphaFold’s precision in structure prediction to PRIME and the success of CataPro in enzyme optimisation, these tools are establishing a foundation for programmable, de novo protein engineering [32,42]. The ongoing shift toward interpretable, multimodal, and dynamic models promises to make the computational design of functional biomolecules a routine reality in synthetic biology and biotechnology.

### 2.3. Generative Models

Enzymes are nature’s most proficient catalysts, orchestrating biochemical transformations with remarkable efficiency and specificity. Yet, the complexity of sequence–structure–function relationships continues to obscure rational enzyme engineering [48]. The advent of generative AI provides new tools to explore these intricate landscapes by learning from massive datasets of natural proteins and generating functional variants de novo. Generative models such as variational autoencoders (VAEs) and generative adversarial networks (GANs) have emerged as powerful frameworks to create enzymes with enhanced catalytic properties, stability, or substrate specificity [49,50]. However, recent studies highlight that these models often generate computational artefacts: sequences that appear plausible in silico but fail to fold or function when experimentally tested, emphasising the need for rigorous validation.

VAEs encode protein sequences into low-dimensional latent representations, enabling interpolation between known variants and the synthesis of plausible new sequences. For instance, MSA-VAE has successfully generated bacterial luciferase (LuxA) variants, 48 of which retained measurable enzymatic activity [51]. Similarly, Conditional VAEs (CVAEs) have been applied to generate functional Cre-like recombinases with predefined DNA-binding specificities, demonstrating the model’s capacity to integrate functional constraints into design [52]. On the other hand, ProteinGAN, employing an adversarial architecture, has produced catalytically active malate dehydrogenase variants, validating GANs as a robust framework for enzyme innovation [53]. Yet, the fraction of active variants is typically low, reflecting the tendency of generative models to produce non-functional design artefacts despite high computational confidence.

#### 2.3.1. Protein Language Models: Learning the Grammar of Life

Parallel to VAEs and GANs, transformer-based protein language models (pLMs) such as ProGen, ESM-2, and ZymCTRL have revolutionised enzyme design by treating amino acid sequences as “biological language” [54,55,56,57,58]. Trained on tens of millions of natural sequences, these models learn residue dependencies and capture the evolutionary syntax underlying protein function. ProGen demonstrated that syntactically coherent sequences can encode catalytically competent enzymes, producing variants of lysozymes and esterases comparable in activity to natural counterparts. ESM-2, trained on over 65 million sequences, further integrates structural understanding, accurately predicting residue–residue contacts and folding patterns to guide functional sequence generation [35]. Nevertheless, even state-of-the-art protein language models frequently assign high confidence to sequences that do not fold experimentally, indicating that computational artefacts remain a significant challenge.

In enzyme-specific applications, ZymCTRL conditions protein generation on Enzyme Commission (EC) numbers, allowing direct synthesis of enzymes with user-specified catalytic classes [59]. Similarly, ProteinMPNN and Fold2Seq use message-passing and transformer embeddings to translate structural information into viable sequences, offering structure-guided de novo design capabilities [60]. However, these systems also produce non-functional design artefacts, requiring extensive downstream screening.

#### 2.3.2. Toward Predictive and Sustainable Enzyme Engineering

The integration of generative AI with biochemical and structural datasets is reshaping enzyme engineering, especially in the context of green chemistry and renewable energy. Generative models can predict how sequence mutations alter catalytic efficiency, thermal stability, or substrate range, thereby guiding laboratory evolution with precision [50]. In bioenergy research, AI-designed enzymes are being explored to improve lignocellulose degradation, CO_2_ fixation, and biosynthetic efficiency of renewable fuels [56]. Yet, the translation of these designs into industrial practice depends on eliminating computational artefacts through iterative experimental validation.

Moreover, the combination of diffusion models and graph neural networks, such as DiffBP and gcWGAN, enables 3D molecular generation and active-site optimisation, bridging sequence-to-structure-to-function mapping [49]. Because many generated folds remain physically unrealizable, these models require careful interpretation to avoid propagation of non-functional design artefacts. As these approaches mature, the predictive design of enzymes with entirely novel catalytic functions will transition from computational aspiration to laboratory reality.

In conclusion, generative AI has transformed enzyme research by enabling predictive, data-driven design using VAEs, GANs, and protein language models. However, meaningful progress depends on filtering computational artefacts through robust biochemical validation pipelines, ensuring that computational innovation translates into functional molecular engineering across medicine, industry, and environmental biotechnology.

### 2.4. Reinforcement Learning (RL)

Reinforcement learning (RL) has become an increasingly powerful paradigm for enzyme engineering, offering a systematic approach to optimise mutagenesis pathways for desired biochemical properties such as catalytic activity and structural stability. In contrast to conventional random or directed mutagenesis, RL employs iterative feedback to balance exploration, the generation of novel amino acid sequences, and exploitation, the refinement of previously identified beneficial mutations [61].

Recent developments have combined RL with deep learning methodologies to enhance molecular design. Pereira et al. demonstrated that the integration of transformer-based self-attention mechanisms with RL enables the evaluation of individual molecular components within a sequence [62]. This allows the assignment of differential rewards to specific residues or structural motifs, providing fine-grained guidance to the generative model. Consequently, the system can design biomolecules with improved target specificity and functional performance.

In protein optimisation, the µProtein framework [61] combines mutational effect prediction (µFormer) with an RL-guided search algorithm (µSearch) to explore the complex fitness landscape of enzymes. This approach has successfully identified multi-point β-lactamase mutants with up to a 2000-fold increase in catalytic efficiency, illustrating the capacity of RL to extract highly functional variants from large sequence spaces.

Additional contributions have further expanded the applicability of RL in molecular bioscience. Haddad et al. employed latent-space RL using proximal policy optimisation to improve the design of molecules with specific physicochemical attributes [63], while Koch, Duigou and Faulon utilised Monte Carlo Tree Search RL to advance bioretrosynthetic pathway design [64].

Collectively, these studies demonstrate that RL, particularly when combined with self-attention and latent-space representations, offers a robust, interpretable and efficient strategy for guiding enzyme engineering. It provides improved connectivity between sequence variation, structural dynamics and catalytic function, thereby advancing the rational design of biocatalysts.

### 2.5. Quantum Computing

Quantum computing represents an emerging frontier in computational biochemistry, aiming to simulate enzyme-catalysed reactions with unprecedented accuracy for reaction pathway optimisation. By exploiting quantum mechanical principles, quantum algorithms can model complex many-body interactions that classical methods cannot efficiently capture [65]. This capability holds particular promise for elucidating catalytic mechanisms, enhancing enzyme selectivity, and accelerating the rational design of novel biocatalysts.

Andersson et al. emphasised that hybrid quantum–classical computing frameworks will likely dominate near-term applications, enabling the precise simulation of quantum-mechanical systems while leveraging classical computational efficiency [66]. Similarly, Gertig et al. demonstrated the value of in silico catalysis and process optimisation through COSMO-RS-based modelling, exemplified by the catalytic carbamate-cleavage process [67].

Despite current challenges in qubit coherence and computational noise, rapid hardware progress is expected to expand the applicability of quantum approaches in biomolecular product design. Ultimately, as quantum technology matures, it may overcome the long-standing computational bottlenecks of enzyme modelling and enable more accurate, efficient, and sustainable biocatalytic process development.

### 2.6. AI Tools Empowering Enzyme and Protein Engineering

The rapid evolution of AI has revolutionised enzyme and protein engineering, enabling unprecedented precision in structure prediction, functional optimisation, and de novo design. Table 2 compares key AI tools, including AlphaFold, RoseTTAFold, ProGen, and ESM-2, summarising their purpose, inputs, outputs, strengths, limitations, and suitability for novel or orphan proteins. Structure-predictive models excel at 3D folding, while generative sequence models enable exploration of new sequences and functional prioritisation [68,69,70]. The table also highlights differences in interpretability, MSA dependence, and experimental integration, guiding tool selection for specific engineering tasks. A diverse suite of these AI tools now underpins every stage of the enzyme engineering pipeline, from sequence analysis and folding prediction to activity modelling, mutational scanning, and expression assessment.

#### 2.6.1. Structure Prediction and Sequence-Based Models

Traditional structure prediction platforms such as AlphaFold2, RoseTTAFold, and OmegaFold provide atomic-level insights into folding and catalytic site architecture, facilitating rational mutagenesis and redesign. Recent advances, including AlphaFold3, extend these capabilities to dynamic systems, multi-chain assemblies, and complex biomolecular interactions, enabling more accurate modelling of protein–protein, protein–ligand, and protein–nucleic acid complexes [69]. Generative and transformer-based sequence models, including ProGen2, ESM-2, and ProteinMPNN, continue to enable the synthesis of entirely new enzyme sequences with improved catalytic efficiency and stability [35,60,68,69]. Molecular docking frameworks such as DiffDock and GNINA assist in predicting substrate binding and ligand interactions, while tools like DynaMut and DeepMutScan evaluate the structural and functional impacts of mutations. AI-based solubility and toxicity predictors such as NetSolP and ToxinPred2 enhance expression and biosafety profiling for industrial and therapeutic applications (Table 1).

#### 2.6.2. Next-Generation Diffusion and Function-Conditioned Models

Recent advances have introduced third-generation models and diffusion-based frameworks that mark a conceptual shift from static structure prediction toward function-conditioned enzyme design. BoltzDesign1, an inversion of the AlphaFold3-derived Boltz-1 model, enables the design of protein binders and enzymes for diverse molecular targets with minimal computational cost, leveraging atomic-level distogram optimisation to generate robust structures with well-defined energy minima [70]. RFdiffusion2, developed by the Baker Lab, directly scaffolds enzyme active sites from sequence-agnostic functional group placements, overcoming prior limitations in residue-level geometry specification and sequence positioning, and successfully designing active catalysts from diverse catalytic sites [71]. EvoDiff combines evolutionary-scale sequence data with diffusion-based generative models, generating high-fidelity, structurally plausible proteins that are inaccessible to structure-based models, including scaffolds for functional motifs and proteins with disordered regions [72]. SE(3)-Diffusion frameworks, such as FrameDiff, operate over orientation-preserving rigid motions in three-dimensional space to generate novel, functional protein backbones up to 500 amino acids without relying on pretrained structure predictors, providing generalisable solutions beyond known natural proteins [73].

Collectively, these next-generation diffusion and sequence-structure-integrated models provide unprecedented control over enzyme design, allowing the generation of de novo sequences and structures conditioned on functional requirements, catalytic site geometry, and evolutionary priors.

## 3. Advances in AI-Driven Enzyme Engineering

**Figure 3:**
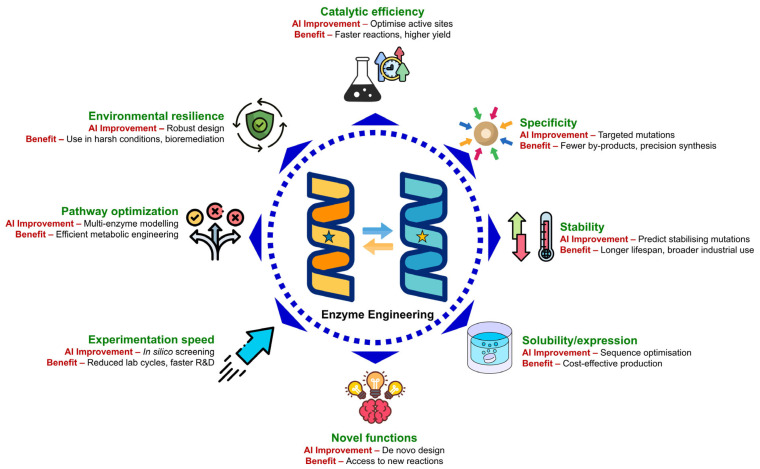
**AI-driven enzyme engineering enhances multiple dimensions of enzyme performance and application efficiency.** AI enables precise prediction and design of enzymes with improved activity, specificity, stability, solubility, and expression. It also accelerates in silico discovery, enables de novo enzyme design, optimises multi-enzyme pathways, and enhances environmental adaptability for industrial and bioremediation applications.

AI is revolutionising enzyme engineering by enabling precise, data-driven optimisation of enzyme properties that were once fine-tuned only through laborious experimental methods. Through advanced machine learning, deep learning, and generative modelling, AI systems can predict, design, and enhance enzyme characteristics with remarkable accuracy. As illustrated in Figure 3, this transformative approach allows scientists to improve catalytic efficiency, substrate specificity, stability, solubility, and even create entirely novel enzymes with desired functionalities. By simulating and predicting enzyme behaviour in silico, AI accelerates discovery cycles, reduces costs, and expands the range of feasible biochemical reactions. As a result, AI-driven enzyme engineering is not only increasing the efficiency and sustainability of industrial bioprocesses but also unlocking next-generation applications in pharmaceuticals, biofuels, synthetic biology, and environmental remediation.

### 3.1. Catalytic Efficiency

The catalytic efficiency of enzymes, primarily quantified by the turnover number (*k_cat_*), constitutes a fundamental determinant of reaction velocity in both biological and industrial contexts. Elevated *k_cat_* values enable more rapid substrate conversion, higher product yields, and reduced operational costs in processes ranging from pharmaceutical synthesis to biofuel production [74,75]. Historically, enhancing catalytic efficiency relied on labour-intensive experimental strategies, such as directed evolution and rational mutagenesis, which are constrained by low throughput, high material costs, and the impracticality of exhaustively exploring large protein sequence spaces [54]. The advent of AI has transformed enzyme engineering into a predictive science, providing computational frameworks that guide mutation selection, substrate optimisation, and enzyme discovery, thereby accelerating the development of highly efficient and robust biocatalysts.

#### 3.1.1. AI Approaches for Predicting Catalytic Efficiency

Machine learning and deep learning methods increasingly provide quantitative predictions of kinetic parameters and mutation effects with high accuracy. Tools such as CataPro integrate molecular fingerprints with pretrained sequence embeddings to predict *k_cat_*, *K_m_*, and catalytic efficiency, enabling the rational identification of high-performance variants and substantially reducing wet-lab screening [42]. Likewise, the ECEP framework combines convolutional neural networks with XGBoost in an ensemble architecture, improving *k_cat_* prediction relative to earlier models (e.g., TurNuP, DLKcat), reducing mean squared error from 0.81 to 0.46 and increasing R^2^ from 0.44 to 0.54 [76]. Generative and embedding-based approaches further expand design capabilities: Xie and Warshel demonstrated that DL-based functional sequence prediction can uncover previously uncharacterised high-turnover variants, accelerating laboratory evolution [48], while DEKP uses graph neural networks and pretrained embeddings to sensitively detect mutation-induced changes in catalytic efficiency [43]. Beyond natural enzymes, platforms such as AI-ZYMES curate nanozyme data and standardise kinetic predictions to streamline discovery and reduce experimental redundancy [77]. Together, these AI-driven approaches enable rational sequence optimisation, guiding modifications that fine-tune active-site geometry, substrate orientation, and turnover without exhaustive empirical screening.

#### 3.1.2. Case Study: AI-Guided Thermostabilisation of Xylanase for Biomass Conversion

AI-guided structure–function modelling was used to enhance the thermostability and catalytic efficiency of the GH11 xylanase PjxA from *Penicillium janthinellum*, a key enzyme in biomass saccharification. AlphaFold2-refined structures, Rosetta stability scoring, and loop-dynamics analysis identified flexible regions near the substrate-binding cleft, predicting that an engineered disulfide bridge would stabilise the active-site architecture. Designed cysteine substitutions were validated by MD simulations, which indicated reduced loop mobility at elevated temperatures. The resulting variant showed markedly improved biochemical performance, increasing the optimum temperature from 50 °C to 70 °C and boosting specific activity by ~4.7-fold, consistent with reported improvements in disulfide-engineered PjxA. When integrated into a saccharification pipeline, the engineered enzyme increased reducing-sugar release by ~38% and enhanced xylose/xylobiose generation from agricultural residues, demonstrating the effectiveness of AI-assisted stabilisation strategies for industrial biomass conversion [78].

#### 3.1.3. Implications and Future Prospects

AI-driven advances are reshaping enzyme engineering from empirical trial-and-error to predictive, mechanism-informed design. Modern frameworks allow accurate forecasting of catalytic efficiency, identification of high-activity variants, and rational mutation prioritisation to optimise *k_cat_* and substrate interactions [77,79]. DL-based generative models and de novo design tools now enable the creation of entirely novel enzymes with tailored functions, while MD simulations and energy-based modelling platforms (e.g., Rosetta) refine predictions by capturing conformational dynamics that influence catalysis [8]. These capabilities minimise experimental burden, accelerate optimisation cycles, and expand biocatalyst applicability in industrial bioprocessing, sustainable manufacturing, synthetic biology, and environmental remediation.

### 3.2. Substrate Specificity and Selectivity

The ability to fine-tune enzyme specificity and selectivity has long been central to advancing biocatalysis, enabling precise transformations in pharmaceuticals, food processing, and green chemistry [80]. Natural enzymes often exhibit high selectivity but limited activity toward non-native substrates, constraining their use in industrial and synthetic contexts [81]. Overcoming these limitations through enzyme engineering, particularly by modulating substrate recognition and catalytic preferences, has been a major focus of biochemical research. The emergence of AI and deep learning is now transforming this field by uncovering complex determinants of enzyme–substrate interactions and enabling rational redesign with unprecedented precision.

#### 3.2.1. AI Approaches for Substrate Specificity Engineering

Early efforts to modify enzyme selectivity relied on directed evolution and rational design. Directed evolution, pioneered in the 1990s, employed iterative cycles of random mutagenesis and high-throughput screening to identify improved variants [82,83]. While successful, this approach was resource-intensive and limited by screening throughput. Rational design used structural and mechanistic knowledge to introduce targeted mutations that alter substrate binding or catalytic outcomes [84].

The integration of ML into enzyme engineering has revolutionised these approaches. Deep learning models can analyse large datasets linking sequence, structure, and function to predict enzyme–substrate compatibility and guide mutational design [85]. High-throughput platforms such as enzyme proximity sequencing (EP-Seq) have generated thousands of sequence–activity pairs for ML model training [86]. These computational insights now enable more precise predictions of residues influencing substrate preference, including allosteric and distal sites.

#### 3.2.2. Case Study: Engineering Monoamine Oxidases for Chiral Drug Intermediates

Engineering monoamine oxidases for chiral pharmaceutical intermediates exemplifies how machine learning–guided biocatalyst development can deliver industrially relevant stereocontrol, as demonstrated in the synthesis of the bicyclic [3.1.0]proline (“P2”) intermediate of the antiviral drug boceprevir. An ML classifier trained on MAO-N variant sequence–activity data was used to predict synergistic mutation sets that reshape the enzyme’s hydrophobic pocket and binding tunnel to preferentially orient the desired (S)-amine intermediate for oxidative desymmetrisation. These predictions informed prioritisation of active-site hotspots for saturation mutagenesis, enabling construction of a sharply reduced, computationally filtered library (<500 variants vs. >10,000 in classical approaches). Subsequent high-throughput screening identified a lead MAO-N variant exhibiting a remodelled access tunnel, a >99% enantiomeric excess, and a 150% improvement in turnover—performance consistent with enhanced MAO-N biocatalysts reported for chemoenzymatic manufacture of P2 via oxidative Strecker chemistry. Industrial deployment of the optimised enzyme in Merck’s workflow further reduced water consumption by ~40% and improved process throughput, supporting a greener and more scalable route to this high-value antiviral intermediate [87].

#### 3.2.3. Implications and Future Prospects

ML-driven enzyme engineering enables predictive, data-guided design, moving beyond empirical trial-and-error. By capturing non-linear relationships between sequence and catalytic performance, models can propose mutations that maximise efficiency and selectivity while minimising undesired activity [88,89]. Integration of AI with molecular dynamics and quantum simulations deepens understanding of how conformational flexibility and transition-state complementarity shape selectivity [85]. In synthetic biology, engineered enzymes with orthogonal specificities allow controlled metabolic networks, and in pharmaceuticals, they enable efficient, enantioselective synthesis of chiral drugs with reduced environmental impact [86]. As datasets of enzyme–substrate interactions grow, de novo design of biocatalysts with programmable specificity is increasingly realistic. The synergy between AI prediction, structural biology, and experimental validation is driving the field toward intelligent, precision biocatalysis, where substrate selectivity is a product of design, not chance.

### 3.3. Stability in Extreme Milieu (Thermal and Extreme pH)

The study of extremostable proteins (or enzymes), biomolecules capable of retaining structure and function under harsh physicochemical conditions, has transformed our understanding of molecular resilience and its industrial potential. Enzymes derived from extremophiles display exceptional stability at high temperatures, extreme pH, salinity, or solvent concentrations. Understanding the multifactorial mechanisms underlying such stability can enable their rational exploitation for industrial biocatalysis, where enzymes must perform efficiently under demanding process conditions [90,91,92,93,94]. With the advent of AI and data-driven methodologies, the frontier of protein engineering has shifted from descriptive to predictive, offering unprecedented opportunities for designing robust enzymes tailored for industrial applications.

#### 3.3.1. Mechanistic Basis of Extremostability and AI Innovations

Early investigations into extremostable proteins revealed that stability arises from multiple synergistic adaptations, including enhanced hydrophobic packing, tighter hydrogen bonding, reduced loop flexibility, and specific amino acid substitutions [94]. Thermophilic enzymes, for example, often display increased main chain hydrogen bonds, aromatic–aromatic interactions, and gamma turns, while reducing residues prone to deamidation such as glutamine and asparagine [90]. No universal rule defines extremostability; it results from a complex interplay between sequence, structure, and environmental context.

Machine learning has emerged as a powerful approach to decode these multifactorial determinants. Early frameworks such as Support Vector Machines (SVM) and Random Forests showed that amino acid composition can outperform nucleotide or structural data in predicting thermostability, achieving accuracies above 90% [94]. More recently, deep learning models, such as Pro-PRIME and ProGen, have enabled the identification of stabilising mutations even in proteins not represented in training datasets [95,96]. Frameworks such as MEnTaT [97] and iCASE [98] leverage evolutionary and physicochemical data to predict stabilising mutations that surpass traditional consensus-based approaches. Integration of high-throughput datasets with ML architectures allows capture of epistatic effects, revealing non-linear interactions among mutations that influence stability [99]. Combining ML predictions with molecular dynamics and stability scoring systems guides targeted mutagenesis by pinpointing flexible or unstable regions [100,101].

#### 3.3.2. Case Study: Designing Alkali- and Heat-Tolerant Cyanide Hydratases

Engineering alkali- and heat-tolerant cyanide hydratases illustrates how computationally guided protein design can enhance enzyme stability in extreme industrial environments. Detoxification of alkaline effluents requires enzymes that remain active at pH > 11, a challenge for wild-type *Bacillus pumilus* cyanide dihydratase (CynDpum). A gradient-boosted ML model predicted mutations that increase surface charge density and reduce loop fraying under high-pH conditions, guiding prioritisation of sites for Rosetta ΔΔG calculations and molecular dynamics simulations. Selected mutations, including E35K, E327G, and Q86R, were experimentally validated, yielding an engineered enzyme that retained more than 90% activity at pH 11 and showed improved thermostability at 42 °C, consistent with previously reported alkali-tolerant CynD variants [102,103]. When deployed in bioreactors treating mining wastewater, the optimised enzyme maintained activity for 72 h, reducing cyanide concentrations below regulatory limits, which was unattainable with the wild-type enzyme, demonstrating the potential of ML-assisted design to achieve robust and industrially viable biocatalysts.

#### 3.3.3. Implications and Future Prospects

AI-driven stability engineering has profound implications for both scientific understanding and industrial innovation. The ability to design enzymes that remain active across broad temperature and pH ranges reduces process costs, enhances scalability, and supports eco-efficient biocatalysis [91,104]. ML enables prediction of stability–activity trade-offs, identification of pH-adaptive residues, and rational selection of combinatorial mutations with synergistic benefits [96].

Challenges remain, including limited data for extreme-condition enzymes, interpretability of deep learning predictions, and the need for iterative feedback between computational models and experimental validation [99]. Future research should focus on unified data standards, model explainability, and integration of molecular dynamics descriptors into ML pipelines. The convergence of extremostability research and AI allows exploration of vast mutational landscapes with precision, uncovering design principles that bridge natural evolution and synthetic innovation, and promising the next generation of resilient, eco-efficient enzymes for industrial biotechnology.

### 3.4. Solubility and Expression Efficiency

Recombinant protein expression is fundamental to biotechnology, yet achieving high solubility and yield remains a persistent challenge, particularly for difficult-to-express proteins (DEPs) [105]. Insoluble or aggregated proteins limit downstream applications in biocatalysis, therapeutics, and diagnostics. Traditionally, solubility has been improved by empirical strategies such as codon optimisation, chaperone co-expression, or fusion tags [104,106]. For instance, fusion with intrinsically disordered tags like NEXT significantly enhanced the solubility and thermostability of *Thermovibrio ammonificans* carbonic anhydrase [106]. However, these approaches are labour-intensive, case-specific, and often fail to generalise across different proteins. To overcome these limitations, ML has emerged as a transformative tool for optimising enzyme solubility and expression efficiency.

Recent ML models exploit amino acid sequence features, physicochemical properties, and evolutionary information to predict protein solubility with high precision. Han et al. developed regression-based predictive models using continuous solubility values rather than binary classifications, achieving an R^2^ of 0.41 and allowing finer discrimination between variants [107]. Similarly, Hirose and Noguchi introduced ESPRESSO, a computational tool that estimates expression and solubility probabilities for *E. coli* and wheat germ systems directly from sequence data [108]. Machine learning-guided cell-free expression systems have also accelerated optimisation cycles, as demonstrated by Landwehr et al., enabling parallel evaluation of over 10,000 enzyme reactions and improving productivity eightfold compared to conventional methods [6].

#### 3.4.1. AI-Guided Mutational Design for Solubility Enhancement

Machine learning provides a powerful strategy to propose targeted mutations that improve protein solubility without compromising catalytic function. Deep learning models trained on large datasets of soluble and insoluble protein variants capture subtle sequence–solubility relationships beyond human intuition. Predictors such as ProteinSol and DeepSol analyse sequence embeddings to forecast solubility changes following point mutations, guiding rational protein redesign, and achieving over 75% accuracy for industrial enzymes and therapeutic proteins [109,110]. Generative ML approaches, including variational autoencoders (VAEs) and protein language models such as ESM-2, can design de novo enzyme sequences optimised for both solubility and expression. Coupled with high-throughput screening frameworks like PUSDA, these approaches explore millions of variant–property pairs, dramatically accelerating the discovery of well-expressed, soluble biocatalysts for sustainable biomanufacturing [111].

#### 3.4.2. Case Study: AI-Enabled Design of Detergent Lipases

Engineering detergent-compatible lipases illustrates how AI-guided design can optimise solubility, expression, and functional performance in surfactant-rich environments. Detergent enzymes must remain soluble and active under alkaline conditions and in the presence of surfactants, which often promote aggregation. A transformer-based solubility model identified hydrophobic surface patches in *Bacillus subtilis* lipase (bsl) that contribute to aggregation, and in silico mutagenesis proposed surface substitutions that increased hydrophilicity while preserving the catalytic Ser–His–Asp triad. The resulting engineered variant, bsl_the3, exhibited improved solubility and surfactant tolerance, retaining approximately 70–80% activity after 24 h at pH 9–10, with enhanced thermostability up to 60 °C. Immobilisation on functionalized ZnO nanoparticles further improved operational stability, maintaining 78–80% activity after 20 reuse cycles or 60 days of storage. Incorporation into prototype detergent formulations increased oil and grease stain removal efficiency by 25–90%, demonstrating that AI-assisted solubility engineering combined with nanoparticle immobilisation can yield robust, industrially viable biocatalysts for high-performance detergent applications [112].

#### 3.4.3. Implications and Future Prospects

Integrating ML with ultra-high-throughput screening enables predictive solubility and activity modelling at an unprecedented scale. ML-driven analysis can navigate trade-offs between solubility and catalytic efficiency, identifying mutations that maintain both properties [111]. These computationally guided designs facilitate scalable, cost-effective enzyme production and accelerate biocatalyst discovery for renewable chemical synthesis [113]. AI-assisted solubility optimisation transforms protein expression from an empirical bottleneck into a data-driven, predictive science.

### 3.5. Novel Enzyme Functions (De Novo Design)

Enzymes are the cornerstone of biological catalysis and industrial biotechnology, enabling the sustainable production of chemicals, fuels, and pharmaceuticals. However, many valuable reactions, such as carbon–fluorine bond cleavage, carbon–silicon bond formation, or non-natural oxidations, are not catalysed by any known natural enzyme. To overcome this limitation, de novo enzyme design seeks to create entirely new proteins capable of performing reactions absent in nature [114]. Recent advances in AI and computational power have transformed this field, enabling the rapid generation of protein sequences predicted to fold into stable and functional enzymes [115]. Nevertheless, it is increasingly recognised that many AI-generated designs exhibit computational artefacts, producing structures that appear plausible in silico but fail to fold or function experimentally, underscoring the need for rigorous biophysical and biochemical validation.

#### 3.5.1. AI in De Novo Enzyme Design

Traditional enzyme engineering via directed evolution or rational design is limited by existing natural scaffolds and mutational search space. In contrast, AI-driven de novo design constructs enzymes from scratch, predicting sequence, structure, and catalytic function simultaneously [116]. Generative AI models such as GENzyme [117] and Riff-Diff [118] exemplify this approach: GENzyme generates enzyme structures conditioned on desired reactions, producing realistic enzyme–substrate complexes [117], while Riff-Diff combines diffusion models and atomistic simulations to scaffold catalytic tetrads, enabling retro-aldol reactions with efficiencies comparable to natural enzymes [118]. However, despite these achievements, generative models often overestimate structural validity, and many predicted scaffolds collapse or misfold when tested experimentally, highlighting the persistent gap between computational confidence scores and true folding stability.

Beyond traditional biocatalysis, the frontier of AI-enabled enzyme design now extends to genome-editing systems. CRISPR-associated nucleases such as Cas9, Cas12, and Cas13 are enzymes whose optimisation heavily relies on AI-guided modelling of sequence–function relationships. Recent breakthroughs, including OpenCRISPR-1 (Confluent, 2024), designed using large language models trained on over one million CRISPR operons [119], and Evo’s de novo Cas enzyme (Arc, 2025), developed through deep multimodal learning across microbial genomes [120], illustrate how AI can re-engineer catalytic specificity, stability, and activity at an unprecedented scale. These advances bridge de novo enzyme design and genome-editing technologies, underscoring AI’s expanding role in both industrial biocatalysis and precision genetic engineering. Yet even in this domain, AI-generated nucleases require extensive iterative refinement because initial designs frequently suffer from poor folding, aggregation, or loss of catalytic activity despite strong in silico predictions.

#### 3.5.2. Case Study: Ferric Enterobactin Esterase Syn-F4, an AI-Designed De Novo Synzyme

The de novo protein Syn-F4 demonstrates that synthetic enzymes can perform life-sustaining reactions using structures and mechanisms distinct from natural enzymes. Isolated from a combinatorial library, Syn-F4 hydrolyses ferric enterobactin, enabling growth of a Δfes *Escherichia coli* strain under iron-limited conditions. Structural analysis revealed a dimeric 4-helix bundle with loops at one end and a central penetrated hole forming a putative active site. Mutagenesis identified Glu26, His74, Arg77, Lys78, and Arg85 as essential, supporting a catalytic dyad mechanism (Glu26–His74). Molecular dynamics and docking confirmed dynamic substrate interactions, providing mechanistic insight into recognition and catalysis. The overall fold and active-site architecture differ from native enterobactin esterases, showing that de novo proteins can achieve biologically relevant catalysis through novel structural solutions. This work illustrates the potential of synthetic biology and AI-guided design to expand enzymatic function beyond natural evolution [121]. At the same time, Syn-F4 highlights a broader trend: only a small fraction of AI-designed candidates achieve such success, making experimental screening essential to distinguish genuine functional designs from computational artefacts.

#### 3.5.3. Implications and Future Prospects

AI-guided de novo enzyme design opens new frontiers in synthetic chemistry, green fuel production, and environmental remediation [115]. Integration of ML-guided optimisation with automated in vivo screening [122] accelerates discovery and validation of superior biocatalysts. De novo design also expands functional diversity in food biotechnology [123]. As AI models such as AlphaFold3, Chai-1, and RFDiffusion evolve, their combination with quantum mechanics and molecular dynamics will further refine active-site prediction and transition-state stabilisation. Future progress will depend on addressing this challenge by integrating experimental feedback loops, uncertainty quantification, and physics-aware modelling to more reliably translate AI-generated designs into functional enzymes. The synergy of deep learning, reaction-aware modelling, and autonomous experimentation brings the goal of designing bespoke enzymes for any reaction within reach, potentially redefining biocatalysis and molecular evolution [124].

### 3.6. Reduced Experimentation Time

Optimising enzymatic reactions traditionally involves labour-intensive and time-consuming trial-and-error experimentation. Each cycle of mutagenesis, screening, and characterisation can span weeks or months, with large volumes of reagents consumed and limited scalability. The advent of ML and AI has transformed this landscape by reducing experimentation time through in silico prediction and data-driven optimisation. These technologies predict the most promising mutations or experimental conditions before laboratory testing, drastically decreasing the number of required experiments, reagents, and costs while accelerating biocatalyst discovery and production [125,126].

#### 3.6.1. AI Approaches to Reduce Experimentation Time

Machine learning has become a powerful tool for navigating the high-dimensional parameter space of enzymatic reactions. In a recent study, Siedentop et al. used Bayesian optimisation (BO) based on Gaussian process regression (GPR) to fine-tune enzyme cascade parameters without experimental replicates [127]. Their approach doubled the productivity-cost ratio while requiring only 52 experiments, demonstrating that algorithmic uncertainty quantification can replace conventional replication strategies. Similarly, Putz et al. introduced a self-driving laboratory (SDL) platform that autonomously conducted and analysed experiments across five-dimensional enzyme–substrate spaces [128]. After 10,000 simulated optimisation campaigns, the SDL identified the most efficient algorithm for reaction optimisation, achieving rapid, data-informed discovery with minimal human input.

Beyond optimisation, microdroplet and AI-assisted screening systems further enhance efficiency. Gantz et al. combined ultra-high-throughput microdroplet screening with ML-based interpretation of 17,000 enzyme variants, achieving up to a 23-fold increase in catalytic rate by testing only a handful of designed mutants [129]. Computational platforms such as FuncLib and Rosetta similarly guide researchers toward beneficial multi-point mutations, avoiding futile screening of neutral variants.

#### 3.6.2. Case Study: ML-Accelerated Discovery of Transaminases for Sitagliptin

The industrial synthesis of the antidiabetic drug sitagliptin traditionally required thousands of enzyme variants through directed evolution to achieve the desired R-enantioselective amine formation. Machine learning minimised experimental effort by training a supervised model on transaminase mutational datasets to predict optimal mutations for the target substrate. Approximately 100 computationally prioritised variants were constructed, and the top mutant achieved 99.95% enantiopurity, in line with previous Codexis/Merck transaminase engineering efforts [130,131]. The engineered enzyme became central to Merck’s green manufacturing process, eliminating heavy-metal catalysts and reducing waste by 19%, while enabling efficient, scalable, and environmentally benign production of sitagliptin. This case exemplifies how ML-guided enzyme design accelerates discovery, reduces experimentation time, and supports sustainable industrial biocatalysis.

#### 3.6.3. Implications and Future Prospects

The integration of AI and ML in enzyme optimisation signifies a paradigm shift from empirical exploration to predictive biocatalyst design. Reduced experimentation time not only enhances sustainability, through savings in materials and energy, but also accelerates industrial bioprocesses, pharmaceutical synthesis, and environmental bioremediation [4]. As AI-driven platforms mature, combining in silico screening, automation, and uncertainty-aware optimisation will enable continuous, self-improving workflows. This fusion of computation and experimentation promises to deliver tailored enzymes with unprecedented speed and precision, redefining the future of biocatalysis and enzyme engineering.

### 3.7. Multi-Enzyme Pathway Optimisation

Metabolic engineering aims to reprogramme cellular networks to enhance the biosynthesis of valuable compounds such as fuels, pharmaceuticals, and fine chemicals [132]. However, heterologous pathway reconstruction often results in flux imbalances, toxic intermediate accumulation, and metabolic burden on the host chassis, limiting productivity [133]. Multi-enzyme pathway optimisation thus emerges as a critical strategy to enhance substrate channelling, mitigate kinetic bottlenecks, and improve cofactor balance [134]. Enzyme co-localisation on synthetic scaffolds or within engineered organelles has been shown to enhance efficiency, exemplified by improved yields in microbial production of antibiotics and terpenoids [134,135].

#### 3.7.1. AI-Guided Frameworks for Pathway Optimisation

Recent innovations have focused on ML-guided frameworks for pathway optimisation. ML algorithms model complex interactions between enzymes and predict flux bottlenecks, enabling rational tuning of enzyme expression levels [136,137]. For instance, Landwehr et al. demonstrated an ML-guided, cell-free platform that optimised amide synthetases across over 10,000 reactions, achieving up to 42-fold activity improvement [6]. Similarly, Xu et al. integrated thermodynamic and enzyme efficiency constraints into metabolic models to enhance flux predictions, leading to a 292% increase in precision over classical stoichiometric approaches [138].

#### 3.7.2. Case Study: AI-Modelled Biosynthetic Pathway for Oligosaccharide Production

Optimising sequential enzyme cascades is challenging due to kinetic bottlenecks and pathway inefficiencies. An AI-driven pathway optimiser, integrating retrosynthetic analysis and kinetic modelling, evaluated over 10^6^ enzyme combinations for lignocellulose conversion, predicting an optimal cascade incorporating a chimeric mannanase–xylanase. This design leveraged prior evidence that bifunctional or chimeric enzymes can enhance substrate deconstruction and catalytic synergy. Experimental implementation of the AI-recommended cascade released approximately 45% more reducing sugars compared with non-optimised pathways, demonstrating improved flux through the pathway. Pilot-scale validation confirmed that the engineered enzyme cocktail not only increased oligosaccharide yield but also reduced reaction time by 30%, highlighting the potential of AI-guided multi-enzyme design to accelerate industrial bioprocessing and maximise functional carbohydrate production [139].

#### 3.7.3. Implications and Future Prospects

AI-driven pathway optimisation provides a transformative route for biomanufacturing chemicals, fuels, and therapeutics. By integrating computational enzyme engineering with ML-guided pathway modelling, predictive analytics can replace empirical trial-and-error, increasing efficiency, scalability, and sustainability [113,136].

### 3.8. Environmental Adaptability

Increasing contamination of ecosystems by plastics, pharmaceuticals, and halogenated xenobiotics necessitates robust biocatalysts capable of functioning under harsh conditions. Enzymes tolerant to solvents, salinity, or toxic substrates are pivotal for bioremediation and sustainable industrial processes [140]. Directed evolution and computational approaches such as FRESCO and FuncLib have enhanced stability and substrate scope of enzymes, including cutinases, PETases, cytochrome P450s, and dehalogenases, enabling the degradation of recalcitrant pollutants like PFAS, TCP, and HCH [141,142,143,144,145].

#### 3.8.1. AI for Environmental Biocatalysis

Machine learning and deep learning frameworks accelerate enzyme optimisation by predicting sequence–function relationships and improving catalytic resilience under environmental stress [140]. ML-guided biodesign, often integrated with metagenomics, has enabled the discovery of extremozymes capable of degrading organophosphates and halogenated agrochemicals, expanding the repertoire of environmentally adaptable enzymes [91,146,147,148].

#### 3.8.2. Case Study: Engineering OP-Degrading Lactonases for Ecological Deployment

The hyperthermostable lactonase SsoPox has been engineered to degrade a broad spectrum of organophosphorus (OP) compounds, demonstrating both catalytic efficiency and environmental robustness. Using a structure-guided, multi-objective design approach, substitutions V27A, Y97W, L228M, and W263M were introduced to enhance core packing, fold rigidity, and active-site flexibility, resulting in the variant SsoPox-αsD6 with a melting temperature of 82.5 °C and broad pH tolerance [149]. This engineered enzyme exhibited substantial increases in catalytic efficiency against multiple OP substrates, including methyl-parathion, malathion, ethyl-paraoxon, and fensulfothion, supporting the role of active-site loop dynamics in broadening substrate specificity. Field-relevant validation in freshwater planarians (Schmidtea mediterranea) confirmed ecological functionality, as SsoPox-αsD6 significantly reduced mortality and improved mobility following pesticide exposure [150]. The variant was further integrated into an immobilised enzyme-based filtration system for water decontamination, demonstrating rapid OP hydrolysis under environmentally variable conditions. This study highlights how rational engineering combined with evolutionary algorithms can produce environmentally adaptable enzymes suitable for in situ bioremediation of toxic organophosphorus compounds.

#### 3.8.3. Implications and Future Prospects

Integrating AI-driven enzyme design with synthetic biology promises to expand accessible chemical space and enable scalable bioremediation. Resilient enzymes such as fungal P450s, peroxidases, and laccases exhibit broad xenobiotic adaptability, including PFAS oxidation [143,144,145,151]. Coupled with techno-economic and life-cycle considerations, these approaches support circular, low-carbon bioprocessing and environmentally sustainable industrial frameworks [140].

## 4. Next-Generation Applications of AI-Engineered Enzymes

The demand for enzymes with enhanced activity, specificity, and stability continues to increase across multiple industrial sectors. Enzyme engineering, supported by rational design, directed evolution, and semi-rational approaches, enables the optimisation of biocatalysts based on structural and mechanistic insights [152]. Artificial intelligence and machine learning accelerate this process by predicting beneficial sequence modifications, guiding mutagenesis, and prioritising variants for experimental validation. These technologies enhance the precision, efficiency, and adaptability of enzyme design, enabling transformative applications in pharmaceuticals, food and agriculture, biofuels, environmental biotechnology, and healthcare [153].

### 4.1. Pharmaceutical and Therapeutic Enzymes

AI and ML have significantly advanced pharmaceutical enzyme engineering by enabling rational prediction and optimisation of enzyme activity, substrate specificity, and structural stability. Engineered transaminases, monoamine oxidases, and ketoreductases are widely employed for asymmetric synthesis, providing high enantioselectivity and improved catalytic performance in the production of pharmaceutical intermediates (Table 3). Therapeutic enzymes, including modified plasminogen activators and prodrug-converting kinases, demonstrate that structural optimisation can enhance stability, substrate preference, and clinical efficacy [154,155,156]. AI tools also facilitate the development of diagnostic enzymes with increased specificity and reduced cross-reactivity, improving biosensor performance [157]. Furthermore, AI-based generative models have been applied to the development of therapeutic enzymes. For example, Moderna Therapeutics has employed deep latent variable modelling combined with automated protein library design to identify ornithine transcarbamylase (OTC) variants with enhanced catalytic activity and thermal stability, thereby improving the potency of mRNA therapeutics for rare metabolic disorders [158]. Collectively, these approaches underscore the growing role of AI-guided enzyme engineering in drug synthesis, therapeutic optimisation, and diagnostic innovation.

### 4.2. Food and Agricultural Enzymes

Hydrolytic and oxidative enzymes such as amylases, cellulases, xylanases, proteases, and lipases are essential in food and feed processing for starch conversion, nutrient fortification, and additive production [180]. Native enzymes frequently require enhanced thermostability, pH tolerance, and catalytic efficiency to meet industrial demands. AI-guided engineering enables targeted mutation selection, stability modelling, and identification of functional hotspots, accelerating enzyme optimisation. Techniques such as site-directed mutagenesis, error-prone PCR, and computational modelling have produced thermostable and efficient variants of xylanases, amylases, and pullulanases capable of high-temperature processing. AI-assisted design further enables the creation of multifunctional or chimeric enzymes with improved catalytic synergy, facilitating the efficient degradation of polysaccharides and the production of functional oligosaccharides (Table 3). These innovations contribute to sustainable and more productive food and agricultural bioprocessing.

### 4.3. Laundry and Detergent Enzymes

Modern detergents require enzymes that remain active under extreme conditions, including elevated temperatures, alkaline pH, and the presence of surfactants or oxidising agents [181]. AI-guided protein engineering enables the rational design of lipases and proteases with improved stability, activity, and compatibility with detergent formulations. Directed evolution, rational design, and computational mutagenesis have produced lipase variants with enhanced thermostability, increased catalytic efficiency, and improved tolerance to detergent components [112,171]. Similarly, engineered serine proteases and subtilisins demonstrate higher low-temperature activity, shifted pH optima, and increased thermal resistance, supporting effective stain removal under energy-efficient washing conditions (Table 3). These advancements facilitate the development of high-performance and environmentally responsible detergents.

### 4.4. Biofuel Enzymes

Enzymes play a critical role in the conversion of lignocellulosic biomass and lipids into bioethanol and biodiesel. Industrial applications require biocatalysts that are thermostable, solvent-tolerant, and catalytically efficient [167]. AI and ML tools enable the prediction of stabilising mutations, active site optimisation, and guided evolution, supporting the development of enzymes suited to these demanding conditions. Improvements achieved through AI-assisted design include enhanced thermostability, reduced product inhibition, and greater tolerance to alcohols and organic solvents (Table 3). These optimisations increase the efficiency of saccharification, transesterification, and overall biomass conversion. AI-guided enzyme engineering also contributes to emerging applications such as enzymatic fuel cells by improving catalytic turnover and operational stability [176].

### 4.5. Environmental Bioremediation Enzymes

AI-guided enzyme engineering is increasingly applied to the development of biocatalysts for degrading persistent environmental pollutants. Computational tools facilitate the optimisation of catalytic activity, substrate specificity, and operational stability under challenging environmental conditions [182,183,184]. Engineering of cyanide hydratases, dye-degrading oxidoreductases, polycyclic aromatic hydrocarbon (PAH)-oxidising cytochrome P450s, and organophosphate-degrading lactonases has been supported by ML-assisted directed evolution, rational mutagenesis, and stability modelling (Table 3). These interventions have led to increased alkali tolerance, enhanced thermostability, higher substrate turnover, and improved compatibility with environmental matrices. Integration with immobilisation and nanomaterial strategies further enhances robustness and reusability. Collectively, these advances demonstrate that AI-enabled enzyme engineering provides a scalable and sustainable approach for effective pollutant remediation.

As summarised in Table 3, the integration of AI and ML into enzyme engineering has enabled the precise optimisation of catalytic performance, stability, and substrate specificity across pharmaceuticals, food processing, biofuels, detergents, and environmental bioremediation. These data-driven approaches have transformed traditional biocatalyst development into a predictive, efficient, and sustainable process, paving the way for next-generation industrial biotechnologies.

## 5. Challenges in AI-Driven Enzyme Engineering

The integration of AI and ML into enzyme engineering has significantly advanced biocatalyst design, enabling exploration of vast sequence spaces and generation of novel variants beyond traditional rational design and directed evolution [185]. Despite these advances, substantial challenges remain that limit the broader applicability and reliability of AI-based enzyme design. These challenges can be categorised into four domains: data, enzyme, reaction, and biochemical complexities. Addressing them is critical for the development of robust, generalizable, and experimentally relevant predictive frameworks [186].

### 5.1. Data-Centric Challenges

#### 5.1.1. Data Quality, Standardisation, and Coverage

The reliability of ML models is contingent on high-quality, standardised datasets. Current enzyme datasets are often inconsistent, incomplete, or reported in non-uniform formats, complicating integration and model development [187]. Standardisation of experimental conditions, substrate identities, and kinetic parameters is limited, reducing reproducibility. Unified and curated datasets, such as EnzymeMap and ECREACT, significantly improve predictive performance compared to traditional databases [188,189]. Adopting FAIR (Findable, Accessible, Interoperable, and Reusable) principles and STRENDA guidelines can further enhance dataset interoperability and reproducibility [190,191].

#### 5.1.2. Limited Access to Proprietary Data

Industrial datasets, which often contain high-value kinetic and functional information, remain largely inaccessible. This restricts reproducibility, model benchmarking, and generalizable training. Open-access repositories and MLOps frameworks, including containerised pipelines and continuous integration, are essential to support transparent and scalable model development [192].

#### 5.1.3. Class Imbalance and Negative Dataset Scarcity

Enzyme datasets are skewed toward well-characterised classes, while rare enzymes and reactions are underrepresented, introducing predictive biases [193]. Negative examples—critical for distinguishing functional from non-functional enzyme-substrate pairs—are often absent, requiring artificial generation via random sampling or biochemically informed heuristics [79]. Future strategies should integrate generative modelling, mechanism-aware learning, and active sampling to improve coverage and predictive reliability.

#### 5.1.4. Incomplete Reaction Representation

Inconsistent reaction annotations, missing stereochemistry, atom mappings, or cofactor/contextual information hinder model interpretability and generalizability [194,195]. Mechanism-aware encodings, hypergraph models, and reactant-product alignment methods are promising solutions but are not yet universally adopted [196].

### 5.2. Enzyme-Centric Challenges

#### 5.2.1. Non-Canonical and Promiscuous Functions

Many enzymes exhibit moonlighting or promiscuous activities that are context dependent [197]. Standard datasets often fail to capture these functions, leading to misclassification or underprediction. Multi-label function prediction, incorporating cellular context and protein–protein interactions, is essential to model the full functional repertoire of enzymes.

#### 5.2.2. Unseen or Non-Homologous Sequences

Current ML models, including transformers, CNNs, and GNNs, often generalise poorly to non-homologous or mutant sequences [31]. Embedding-based approaches from large pretrained protein language models show promise, but their robustness in predicting function for distant or engineered variants requires further exploration.

### 5.3. Reaction-Centric Challenges

#### 5.3.1. Multistep Reaction Complexity

Enzyme-driven multistep reactions present combinatorial and mechanistic challenges. Structural dynamics, intermediate stability, cofactor requirements, and pathway compartmentalisation are often neglected, compromising predictive accuracy [198]. Integration of forward reaction prediction with retrosynthetic planning, along with thermodynamic and host-context modelling, is essential to improve reliability.

#### 5.3.2. Prediction of Novel or Uncharacterised Reactions

Predicting enzyme activity for unannotated or non-canonical reactions remains a key challenge. Existing ML models have limited capacity to pair enzymes with new substrates or to predict multistep and promiscuous transformations. Incorporating reactive-site mapping and concatenated sequence-reaction features into transformer-based architecture can enhance the discovery of novel biocatalytic reactions [13].

### 5.4. Biochemical and Contextual Challenges

#### 5.4.1. Limitations of EC Number Classification

The traditional EC system often fails to capture enzyme multifunctionality, context-dependent specificity, and diverse reaction outcomes [199]. Misannotations further hinder accurate predictions [200]. Multi-label GO-based annotations combined with protein embeddings provide a more flexible framework for functional prediction [201].

#### 5.4.2. In Vitro Versus in Vivo Conditions

Kinetic data measured under controlled in vitro conditions may not reflect physiological cellular environments, where metabolite concentrations, protein expression levels, and regulatory networks influence enzyme activity [202]. Standardised collection of in vivo contextual metadata is crucial for physiologically relevant predictive modelling.

#### 5.4.3. Environmental Variability

pH, temperature, cofactor availability, and other environmental factors significantly modulate enzyme kinetics and specificity. Current ML frameworks largely assume static conditions, limiting their predictive scope [13]. Systematic integration of environmental metadata is required for more accurate and generalizable predictions.

#### 5.4.4. Experimental Validation Bottlenecks

Despite high predictive performance, translating ML-generated predictions to validated experimental outcomes is challenging. Limitations include cost, assay design, protein expression hurdles, and substrate availability [13]. Hybrid workflows combining in silico prioritisation with targeted high-throughput screening and standardised validation protocols are necessary to close the design-experiment gap.

**Figure 4:**
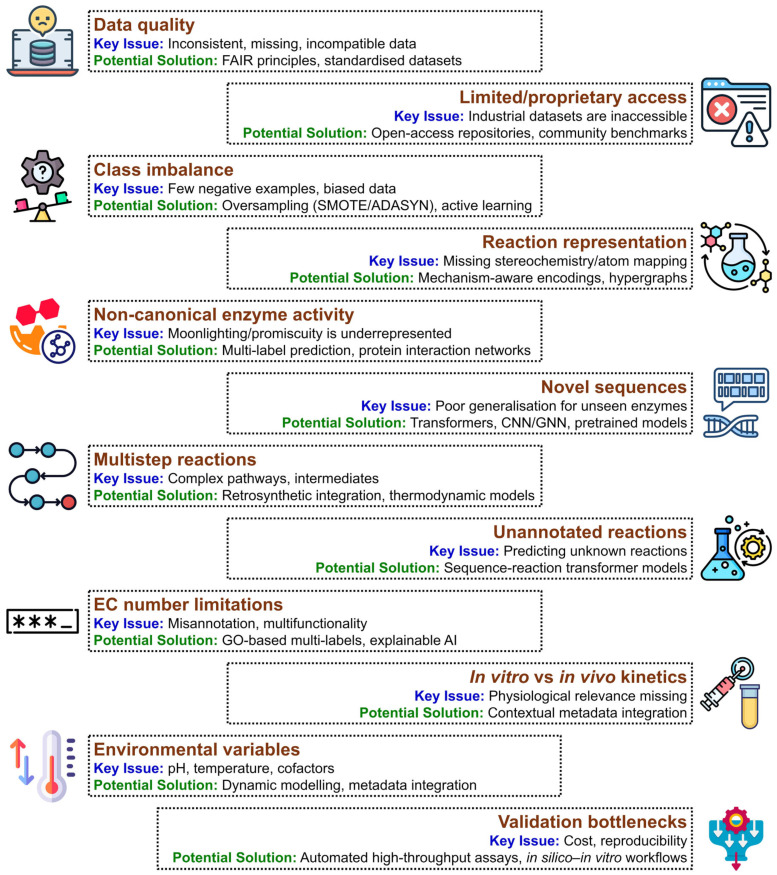
**Challenges and potential solutions in AI applications for enzyme function and reaction prediction.** Key challenges in applying AI to enzyme function and reaction prediction are outlined alongside potential solutions. The schematic highlights issues in data quality, representation, annotation, and validation, with strategies such as FAIR data principles, mechanism-aware modelling, and hybrid in silico–in vitro workflows to enhance model reliability and generalisability.

An overview of these key challenges and corresponding potential solutions is presented in Figure 4.

## 6. Conclusions and Future Directions

AI-driven enzyme engineering represents a transformative advance in how biocatalysts are discovered, optimised, and applied across scientific and industrial domains. By integrating machine learning, deep learning, and generative modelling, researchers can now predict enzyme structure–function relationships, identify mutational hotspots, and design de novo proteins with exceptional precision. Tools such as AlphaFold 2, ESM-2, and ProteinGAN have expanded access to previously unexplored sequence spaces, while hybrid AI and directed evolution strategies have accelerated the discovery of high-performance enzyme variants. These developments are revolutionising sectors such as pharmaceuticals, biofuels, and environmental biotechnology by enabling faster, more sustainable, and cost-effective processes. Although challenges remain in data quality, model interpretability, and experimental validation, the convergence of AI with systems biology, quantum computing, and high-throughput screening holds great promise for overcoming these barriers. AI-driven enzyme engineering is establishing a foundation for the rational, efficient, and scalable creation of next-generation biocatalysts, broadening the scope and impact of modern biotechnology.

Future research should target both technical and conceptual milestones that can drive the next generation of enzyme engineering. A key priority is the creation of standardised, high-quality, and FAIR-compliant datasets that include negative examples, detailed reaction metadata, and consistent molecular and sequence representations to support robust training and evaluation. Advancing predictive accuracy will require hybrid architectures that integrate sequence information, three-dimensional structural data, reaction context, and dynamic conformational ensembles, enabling generalisation across diverse enzyme families. Incorporating multi-label functional annotations, cellular context, and environmental parameters will improve physiological relevance and bridge the gap between computational predictions and experimental outcomes.

Game-changing innovations may emerge from integrating AI with real-time experimental feedback, automated high-throughput platforms, and in situ mutagenesis screening. Ensemble predictors, mechanistic modelling, and explainable AI could provide deeper insight into catalytic mechanisms, allosteric effects, and substrate specificity, moving beyond black-box predictions toward interpretable design. Community-wide benchmarks, reproducible evaluation standards, and open-access model repositories will further accelerate collaborative progress and ensure fair, consistent assessment of emerging methodologies.

Collectively, these strategies will transform AI-driven enzyme engineering into a reliable, generalisable, and predictive platform, enabling not only the rational design of biocatalysts for existing applications but also the exploration of entirely novel catalytic functions. By aligning computational foresight with experimental innovation, the field is poised to achieve unprecedented efficiency, sustainability, and functional versatility in biocatalyst development, ultimately shaping the future of biotechnology, green chemistry, and synthetic biology.

## Figures and Tables

**Table 1:** Examples of AI tools commonly used for enzyme and protein engineering.

Tool	Description	Key Features/Applications	Developer/Source	Tool URL
***Structure Prediction***				
AlphaFold2 (v2.0.0)	Deep learning-based protein structure prediction	High-accuracy 3D folding from sequence data; catalytic site inference	DeepMind	https://www.deepmind.com/research/highlighted-research/alphafold (accessed on 1 October 2025)
RoseTTAFold (v1.0)	Multi-track neural network integrating sequence and structural information	Predicts structure and function; supports novel fold design	Baker Lab	https://boinc.bakerlab.org/rosetta/ (accessed on 1 October 2025)
Chai-1	Enhanced structure prediction using multimodal inputs (MSAs, templates, embeddings)	Robust prediction across diverse biomolecules	Chai Discovery	https://neurosnap.ai/service/Chai-1 (accessed on 1 October 2025)
OmegaFold (v1.1.0)	Structure prediction without multiple sequence alignments (MSAs)	Accurate, MSA-free folding for low-homology sequences	HeliXon Protein Inc.	https://github.com/HeliXonProtein/OmegaFold or https://cosmic-cryoem.org/tools/omegafold/ (accessed on 1 October 2025)
HelixFold	Fast, efficient structure prediction framework	Optimised for industrial biodesign pipelines	PaddleHelix	https://github.com/PaddlePaddle/PaddleHelix/tree/dev/apps/protein_folding/helixfold (accessed on 26 October 2025)
FastFold	Speed-optimised AlphaFold implementation	GPU-parallelisation for rapid inference	ByteDance	https://github.com/hpcaitech/FastFold (accessed on 1 October 2025)
***Protein and Enzyme Design***				
NeuroFold	AI-guided enzyme design platform	Generates and screens enzyme variants with improved catalytic traits	Neurosnap	https://neurosnap.ai/service/NeuroFold (accessed on 1 October 2025)
ProGen2 (v2.0)	Transformer-based language model for protein generation	De novo protein sequence generation preserving function	Salesforce AI Research	https://github.com/salesforce/progen (accessed on 1 October 2025)
ESM-2 (v1.0.0)	Large-scale protein language model	Sequence embeddings, mutation effect, and function prediction	Meta AI (FAIR)	https://github.com/facebookresearch/esm (accessed on 1 October 2025)
RFdiffusion	Diffusion-based generative model for protein backbones	Enables creation of novel folds and catalytic sites	Baker Lab/UW	https://github.com/RosettaCommons/RFdiffusion (accessed on 1 October 2025)
ProteinMPNN (v1.0.0)	Sequence design conditioned on backbone structures	Rapid backbone-to-sequence mapping for design tasks	Baker Lab/UW	https://github.com/dauparas/ProteinMPNN (accessed on 1 October 2025)
Chroma	Controlled generative framework for structural design	Conditional structure generation with user-specified features	Generate:Biomedicines	https://generatebiomedicines.com/chroma (accessed on 26 October 2025)
***Catalytic Site, Substrate Specificity and Metal-Binding Prediction***				
MAHOMES II	RF-based site model	Predicts catalytic metal ions in enzymes	Robinson Lab	https://mahomes.ku.edu/help (accessed on 1 October 2025)
AdenylPred	RF-based classifier	Predicts functional and substrate classes of adenylate-forming enzymes	Robinson Lab	https://github.com/serina-robinson/adenylpred (accessed on 1 October 2025)
innov’SAR	PLSR framework	Predicts turnover, stability, and stereoselectivity	PEACCEL (The AI company for Life Science)	https://www.peaccel.com/technology/innovsar-artificial-intelligence-platform/ (accessed on 1 October 2025)
gcWGAN	Deep generative model	3D active-site and sequence generation	Shen Lab	https://github.com/Shen-Lab/gcWGAN (accessed on 1 October 2025)
***Molecular Docking***				
DiffDock/DiffDock-L	Diffusion-based molecular docking model	Flexible ligand–protein docking; 3D pose generation	Baker Lab/UW	https://github.com/gcorso/DiffDock (accessed on 1 October 2025)
GNINA	Convolutional neural network (CNN)-based docking framework	ML-enhanced scoring and ligand ranking	GNINA Team	https://github.com/gnina/gnina or https://proteiniq.io/app/gnina (accessed on 26 October 2025)
PocketFlow	AI-based pocket prediction and docking	Predicts binding pockets and supports flexible docking	Tencent AI Lab	https://proteiniq.io/app/pocketflow or https://github.com/Saoge123/PocketFlow (accessed on 26 October 2025)
AutoDock Vina	Classical open-source docking program	Widely used for small-molecule and enzyme–ligand interactions	Scripps Research	http://vina.scripps.edu (accessed on 26 October 2025)
**In silico *Mutagenesis***				
DeepMutScan	Predicts the functional impact of mutations	High-throughput mutational scanning via deep learning	Gamazon Lab	https://github.com/gamazonlab/DeepMutScan (accessed on 26 October 2025)
MuPIPR	Predicts protein–protein interaction changes upon mutation	Estimates ΔΔG and interface disruption	Zhou Lab	https://github.com/guangyu-zhou/MuPIPR (accessed on 26 October 2025)
ESM-IF1 (v2.0.1)	Inverse folding model (structure → sequence)	Recovers sequences from 3D backbones	Meta AI	https://neurosnap.ai/service/ESM-IF1 (accessed on 1 October 2025)
DynaMut2 (v2.0)	Predicts mutation effects on stability and dynamics	Visualises conformational and flexibility changes	Biosig Lab	https://biosig.lab.uq.edu.au/dynamut/ (accessed on 26 October 2025)
***Sequence and Structure Analysis***				
ProtNLM/ProtBERT/ESM-1b	Protein language models for representation learning	Embeddings for annotation, classification, and alignment	ProtNLM by Google Research/EBI; ProtBERT by Brandes et al.; ESM-1b by Meta AI/Hugging Face	https://310.ai/docs/function/protnlm (accessed on 1 October 2025); https://huggingface.co/Rostlab/prot_bert (accessed on 26 October 2025); https://huggingface.co/facebook/esm1b_t33_650M_UR50S (accessed on 26 October 2025)
MMseqs2	Protein sequence clustering and alignment	Scalable similarity search and redundancy reduction	MPI for Developmental Biology	https://toolkit.tuebingen.mpg.de/tools/mmseqs2 (accessed on 1 October 2025)
Foldseek	Structure-based search engine	Fast comparison of protein 3D structures	MPI for Biology	https://search.foldseek.com/search (accessed on 1 October 2025)
MAFFT (v7.526)	Multiple sequence alignment algorithm	High-speed, accurate alignment for large datasets	Osaka University	https://mafft.cbrc.jp/alignment/software/ (accessed on 1 October 2025)
HMMER (v3.4)	Hidden Markov Model-based alignment and domain search	Detects conserved motifs and functional domains	HHMI/Sean Eddy	http://hmmer.org (accessed on 1 October 2025)
***Enzymatic Classification and Functional Prediction***				
DeepEC (v1.0)	CNN-based enzymatic classifier	Predicts complete EC numbers from sequence	KAIST Systems Biology	https://bitbucket.org/kaistsystemsbiology/deepec (accessed on 1 October 2025)
ECPred (v1.1)	Ensemble (SVMs, kNN)	Predicts full/partial EC numbers	Alperen Dalkıran	https://ecpred.kansil.org/ (accessed on 1 October 2025)
***Expression and Safety Tools***				
NetSolP (v1.0)	Predicts protein solubility using deep learning	AI-based solubility and expression classifier	DTU Health Tech	https://services.healthtech.dtu.dk/service.php?NetSolP-1.0 (accessed on 1 October 2025)
SoDoPE	Predicts heterologous expression efficiency	Assists codon optimisation and solubility enhancement	SoDoPE Team	https://tisigner.com/sodope (accessed on 1 October 2025)
ToxinPred2 (v2.0)/ADMET-AI (v1.4.0)/eTox (v0.97)	Predicts toxicity, allergenicity, and pharmacokinetics	Comprehensive in silico safety and ADMET profiling	Various Academic Teams	https://webs.iiitd.edu.in/raghava/toxinpred2/ (accessed on 1 October 2025)
***Antibody and Binder Design***				
NeuroBind	AI-based design of antibodies, peptides, and nanobodies	Generates high-affinity binders and optimises stability	Neurosnap Inc.	https://neurosnap.ai/service/NeuroBind (accessed on 1 October 2025)
ABlooper	Deep-learning tool for antibody CDR loop modelling	Rapid and accurate loop conformation prediction	Oxford Protein Informatics Group	https://github.com/oxpig/ABlooper (accessed on 1 October 2025)
IgFold (v0.4.0)	Transformer model for antibody structure prediction	Sequence-to-structure mapping for immunoglobulins	Johns Hopkins University	https://github.com/Graylab/IgFold (accessed on 26 October 2025)
NanoNet	Lightweight nanobody structure predictor	Fast prediction of VHH domains and single-chain binders	Dina Lab	https://github.com/dina-lab3D/NanoNet (accessed on 26 October 2025)

**Table 2:** Comparison of key AI tools for enzyme engineering.

Feature	AlphaFold2 (v2.0.0)	RoseTTAFold (v1.0)	ProGen (v1.0)	ESM-2 (v1.0.0)
***Primary purpose***	High-accuracy 3D structure prediction	Fast protein/complex structure prediction	Generative protein sequence design	Sequence embeddings and functional prediction
***Input***	AA sequence; deep MSA; optional templates	AA sequence; shallow MSA	Sequence context; optional functional/family tags	AA sequence only
***Output***	Atomic 3D structure; pLDDT; PAE	3D structure; confidence metrics	Novel sequences; residue probability profiles	Sequence embeddings; functional scores
***Approach***	Attention-based DL (EvoFormer); MSA-driven	3-track network integrating sequence–distance–coordinates	Autoregressive protein language model	Transformer protein language model
***Accuracy***	Very high for proteins with homologs; moderate for orphan proteins	Good; slightly below AF2; more robust with shallow MSAs	Variable; sequence plausibility high, functional accuracy depends on filtering	High for function/mutation prediction; not for 3D structure
***Usability***	Moderate (requires GPUs and MSA tools); many community pipelines	High; faster and easier to run; less computationally heavy	High; simple interface; no structural prediction required	High; embeddings easy to compute; scalable to large datasets
***Runtime***	Hours per protein (depending on MSA depth)	Minutes to hours; typically faster than AF2	Seconds–minutes for sequence generation	Seconds for embeddings; minutes with downstream predictors
***Availability***	Open-source (DeepMind)	Open-source (Baker lab)	Available via API (Meta/Anthropic; semi-open)	Open-source model weights (Meta)
***Use in enzyme engineering***	Widely adopted for fold validation, active-site inspection, and variant analysis	Increasingly used for cross-validation and modelling complexes	Used in published de novo enzyme design and function-conditioned libraries	Frequently used for mutational scanning, functional prediction, and variant prioritisation
***Strengths***	Near-experimental accuracy; robust confidence metrics	Fast; models complexes; lower MSA burden	Explores sequence space beyond natural diversity	Effective without MSAs; good for orphan proteins; strong functional predictions
***Limitations***	MSA-dependent; static structures; limited dynamics/ligand modelling	Lower accuracy than AF; static structures	No structure prediction; generated sequences may not fold/function	Does not output structures; requires downstream modelling
***Best use cases***	Structure-guided mutagenesis; variant stability screening	Complex modelling; alternative structural predictions	De novo enzyme design; generating diverse sequence libraries	Variant ranking; function prediction; prioritising mutations
***Handling of distant homologs***	Weak when MSA sparse	Slightly more robust than AF2 but still MSA-dependent	Can generate sequences without homologs	Excels at orphan proteins; MSA-independent
***Interpretability***	Medium; confidence metrics aid interpretation	Medium	Low	Low (requires downstream interpretation)
***Integration with experimental workflow***	Strong in structure-based design and active-site mapping	Useful for folding validation and complex modelling	Provides candidates for testing and directed evolution	Guides mutational experiments; prioritises variants
***References***	[32,34,69,70]	[70,71]	[68,72]	[35]

AA—amino acid; MSA—multiple sequence alignment; DL—deep learning; AF2—AlphaFold2; PAE—predicted aligned error; pLDDT—predicted Local Distance Difference Test.

**Table 3:** AI- and ML-driven enzyme engineering applications across industrial sectors.

Enzyme/System	Source	Industrial Purpose	AI/Engineering Strategy	Mutation(s)	Key Performance Improvement	Reference
***Pharmaceuticals and Healthcare***						
R-selective transaminase	*Arthrobacter* sp.	Sitagliptin synthesis	Directed evolution	Multiple rounds (not disclosed)	99.95% enantiopurity	[130,131]
Monoamine oxidase	*Aspergillus niger*	Chiral amines for boceprevir	Directed evolution + computational optimisation	Not specified	150% ↑ yield; 40% ↓ water use	[87]
Ketoreductases (KREDs)	*Lactobacillus kefir*, *Sporobolomyces salmonicolor*	Chiral intermediates (montelukast)	Rational mutagenesis	Not specified	>99.55% enantioselectivity	[159,160]
Transaminases and Monoamine Oxidases	Various microbial sources	Chiral intermediates (imagabalin, etc.)	Computation + directed evolution	Not specified	>97% R-enantiomer yield	[161,162]
Tissue Plasminogen Activator (tPA)/Te-necteplase (TNKase^®^)	Humans	Thrombolytic therapy	Rational engineering	Multiple substitutions	↑ half-life; ↑ inhibitor resistance	[154]
Nucleoside Kinase (HSV-TK/CH1)	Herpes simplex type 1	Prodrug activation	Structure-guided design	Fusion construct	Higher cancer-cell specificity	[154,155]
Fructosyl Peptide Oxidase (FPOX)	*Eupenicillium terrenum*	Diabetes biosensing	AI-guided design	Y261W	↑ specificity; reduced cross-reactivity	[157]
***Food and Agriculture***						
Endo-1,4-β-Xylanase	*Trichoderma reesei*	Thermostable xylanase	Error-prone PCR	T2C, T28C	Stability ↑ from 40 s to 20 min at 65 °C	[163]
GH11 Xylanase	*Neocallimastix patriciarum*	Heat-tolerant hydrolysis	ML (B-factor analysis)	Q87R, N88G, S89H, S90T	50–60% residual activity at 60–65 °C	[164]
α-Amylase	*Thermus thermophilus*, *Bacillus* sp. TS-23	High-temp starch hydrolysis	Directed evolution + site mutagenesis	Multiple	Improved high-temperature stability	[165,166]
Mannanase–Xylanase Chimera	Recombinant system (SpyTag/SpyCatcher)	Biomass breakdown	Isopeptide cyclisation	SpyTag/SpyCatcher fusion	↑ stability; ↑ metal tolerance	[139]
Pullulanase	*Bacillus deramificans*	Syrup production	Site-directed mutagenesis	D437H, D503Y	↑ thermostability	[167]
L-Aspartate α-Decarboxylase/D-Hydantoinase	*Bacillus stearothermophilus*	Amino acid/additive synthesis	Error-prone PCR + modelling	Multiple	2.45–200× ↑ catalytic efficiency	[168,169]
Xylanase (xylose-tolerant mutants)	*Bacillus* spp.	Starch/biofuel processes	ML-guided random mutagenesis	L133V, M116I, L131P	3–3.5× ↑ catalytic efficiency	[170]
***Laundry and Detergent Industry***						
Lipase (eP-231-51 mutant)	*Bacillus licheniformis*	Thermostable detergent lipase	Directed evolution	D72G, D61G, Y129H, T101P	13.5× ↑ thermostability; 30% ↑ activity	[171]
Lipase (bsl_the3 mutant)	*Bacillus subtilis*	Alkaline-stable lipase	Rational design	F41K, W42E, P119E, Q121K, V149K, Q150E	Active at pH 9; ↑ surfactant tolerance	[112]
Alkaline Serine Protease	*Bacillus pumilus* BA06	Cold-active protease	Directed evolution + site mutagenesis	P9S/K27Q; P9S/T162I	5× ↑ activity at 15 °C	[172]
Subtilisin Protease	*Bacillus gibsonii*	High-temperature protease	Charge engineering	N253D, Q256E	↑ thermal resistance; shifted pH optimum	[173]
***Biofuels***						
Xylanase	*Penicillium janthinellum* MA21601	Biomass hydrolysis	Site mutagenesis	Disulfide bridge insertion	Optimum temp 50–70 °C; 4.76× ↑ activity	[77]
Xylanase (xylose-tolerant)	*Bacillus* spp.	Lignocellulose conversion	ML-guided evolution	M116I, L131P, L133V	3× activity; 3.5× ↑ Ki	[170]
Lipase	*Geobacillus stearothermophilus* T6	Biodiesel transesterification	Consensus design + mutagenesis	H86Y, A269T; Q185L	66× ↑ stability; +4.3 °C Tm	[174]
Lipase	*Streptomyces* sp. W007	Triacylglyceride conversion	Site-directed mutagenesis	F153A, H108A, V233A	↑ activity and thermostability	[175]
6-Phosphogluconate Dehydrogenase	*Thermotoga maritima*	Enzymatic fuel cells	Directed evolution	Not disclosed	42× ↑ activity at pH 5.4	[176]
***Environmental Bioremediation***						
Cyanide Dihydratase (CynD)	*Bacillus pumilus*	Cyanide degradation	ML-assisted evolution	E35K, E327G, R322, etc.	↑ alkali tolerance; ↑ thermostability	[102,103]
Ginger Peroxidase (GP)	*Zingiber officinale*	Dye degradation	ML-guided immobilisation	Immobilisation, no sequence change	3× ↑ Vmax; ↑ stability	[177]
Cytochrome P450 (CYP101)	*Pseudomonas putida*	PAH degradation	AI-guided mutagenesis	Y96A; F87A–Y96F	31% ↑ NADH turnover	[178]
Cytochrome P450 (CYP5136A3)	*Phanerochaete chrysosporium*	PAH oxidation	Structure-guided mutagenesis	L324F; W129F/L324F	23–187% ↑ oxidation	[179]
Phosphotriesterase-like Lactonase (SsoPox-αsD6)	*Sulfolobus solfataricus*	OP pesticide degradation	Computational design + mutagenesis	V27A, Y97W, L228M, W263M	Tm = 82.5 °C; ↑ OP detoxification	[149,150]

↑ is increased; ↓ is decreased.

## Data Availability

No new data were created or analyzed in this study. Data sharing is not applicable to this article.

## References

[1] Fasim A., More V.S., More S.S. (2021). Large-scale production of enzymes for biotechnology uses. Curr. Opin. Biotechnol..

[2] Yang J., Li F.Z., Arnold F.H. (2024). Opportunities and challenges for machine learning-assisted enzyme engineering. ACS Cent. Sci..

[3] Mao S., Jiang J., Xiong K., Chen Y., Yao Y., Liu L., Liu H., Li X. (2024). Enzyme engineering: Performance optimization, novel sources, and applications in the food industry. Foods.

[4] Mazurenko S., Prokop Z., Damborsky J. (2019). Machine learning in enzyme engineering. ACS Catal..

[5] Singh N., Lane S., Yu T., Lu J., Ramos A., Cui H., Zhao H. (2025). A generalized platform for artificial intelligence-powered autonomous enzyme engineering. Nat. Commun..

[6] Landwehr G.M., Bogart J.W., Magalhaes C., Hammarlund E.G., Karim A.S., Jewett M.C. (2025). Accelerated enzyme engineering by machine-learning guided cell-free expression. Nat. Commun..

[7] Zhou J., Huang M. (2024). Navigating the landscape of enzyme design: From molecular simulations to machine learning. Chem. Soc. Rev..

[8] Sun R., Wu D., Chen P., Zheng P. (2024). Cutting-edge computational approaches in enzyme design and activity enhancement. Biochem. Eng. J..

[9] Palabiyik A.A. (2025). Synzymes: The future of modern enzyme engineering. Appl. Biochem. Biotechnol..

[10] Chen A., Peng X., Shen T., Zheng L., Wu D., Wang S. (2025). Discovery, design, and engineering of enzymes based on molecular retrobiosynthesis. mLife.

[11] Ferreira P., Fernandes P.A., Ramos M.J. (2022). Modern computational methods for rational enzyme engineering. Chem. Catal..

[12] Markus B., Christian C G., Andreas K., Arkadij K., Stefan L., Gustav O., Elina S., Radka S. (2023). Accelerating biocatalysis discovery with machine learning: A paradigm shift in enzyme engineering, discovery, and design. ACS Catal..

[13] Tripathi N., Hérisson J., Faulon J.L. (2025). Machine learning in predictive biocatalysis: A comparative review of methods and applications. Biotechnol. Adv..

[14] Hanna C., Blot A., Petke J. (2025). Reinforcement learning for mutation operator selection in automated program repair. Autom. Softw. Eng..

[15] Feehan R., Montezano D., Slusky J.S. (2021). Machine learning for enzyme engineering, selection and design. Protein Eng. Des. Sel..

[16] Kouba P., Kohout P., Haddadi F., Bushuiev A., Samusevich R., Sedlar J., Damborsky J., Pluskal T., Sivic J., Mazurenko S. (2023). Machine learning-guided protein engineering. ACS Catal..

[17] Thomas N., Belanger D., Xu C., Lee H., Hirano K., Iwai K., Polic V., Nyberg K.D., Hoff K.G., Frenz L. (2025). Engineering highly active nuclease enzymes with machine learning and high-throughput screening. Cell Syst..

[18] Liu S.H., Bai L., Wang X.D., Wang Q.Q., Wang D.X., Bornscheuer U.T., Ao Y.F. (2025). Machine learning-guided protein engineering to improve the catalytic activity of transaminases under neutral pH conditions. Org. Chem. Front..

[19] Camacho C., Coulouris G., Avagyan V., Ma N., Papadopoulos J., Bealer K., Madden T.L. (2009). BLAST+: Architecture and applications. BMC Bioinform..

[20] Sigrist C.J., De Castro E., Cerutti L., Cuche B.A., Hulo N., Bridge A., Bougueleret L., Xenarios I. (2012). New and continuing developments at PROSITE. Nucleic Acids Res..

[21] Finn R.D., Coggill P., Eberhardt R.Y., Eddy S.R., Mistry J., Mitchell A.L., Potter S.C., Punta M., Qureshi M., Sangrador-Vegas A. (2016). The Pfam protein families database: Towards a more sustainable future. Nucleic Acids Res..

[22] Shen H.B., Chou K.C. (2007). EzyPred: A top–down approach for predicting enzyme functional classes and sub-classes. Biochem. Biophys. Res. Commun..

[23] De Ferrari L., Aitken S., van Hemert J., Goryanin I. (2012). EnzML: Multi-label prediction of enzyme classes using InterPro signatures. BMC Bioinform..

[24] Li Y., Wang S., Umarov R., Xie B., Fan M., Li L., Gao X. (2018). DEEPre: Sequence-based enzyme EC number prediction by deep learning. Bioinformatics.

[25] Dalkiran A., Rifaioglu A.S., Martin M.J., Cetin-Atalay R., Atalay V., Doğan T. (2018). ECPred: A tool for the prediction of the enzymatic functions of protein sequences based on the EC nomenclature. BMC Bioinform..

[26] Zou Z., Tian S., Gao X., Li Y. (2019). mlDEEPre: Multi-functional enzyme function prediction with hierarchical multi-label deep learning. Front. Genet..

[27] Ryu J.Y., Kim H.U., Lee S.Y. (2019). Deep learning enables high-quality and high-throughput prediction of enzyme commission numbers. Proc. Natl. Acad. Sci. USA.

[28] Song J., Li F., Takemoto K., Haffari G., Akutsu T., Chou K.C., Webb G.I. (2018). PREvaIL, an integrative approach for inferring catalytic residues using sequence, structural, and network features in a machine-learning framework. J. Theor. Biol..

[29] Torng W., Altman R.B. (2019). High precision protein functional site detection using 3D convolutional neural networks. Bioinformatics.

[30] Ao Y.F. (2025). Machine learning-assisted protein engineering for improving stereoselectivity. Chem. Catal..

[31] Li G., Qin Y., Fontaine N.T., Chong M.N.F., Maria-Solano M.A., Feixas F., Cadet X.F., Pandjaitan R., Garcia-Borràs M., Cadet F. (2021). Machine learning enables selection of epistatic enzyme mutants for stability against unfolding and detrimental aggregation. ChemBioChem.

[32] Casadevall G., Duran C., Osuna S. (2023). AlphaFold2 and deep learning for elucidating enzyme conformational flexibility and its application for design. JACS Au.

[33] Chen B., Khan M.T., Goussetis G., Sellathurai M., Ding Y., Mota J.F. (2025). COMET: Co-Optimization of a CNN Model using Efficient-Hardware OBC Techniques. arXiv.

[34] Jumper J., Evans R., Pritzel A., Green T., Figurnov M., Ronneberger O., Tunyasuvunakool K., Bates R., Žídek A., Potapenko A. (2021). Highly accurate protein structure prediction with AlphaFold. Nature.

[35] Lin Z., Akin H., Rao R., Hie B., Zhu Z., Lu W., Smetanin N., Verkuil R., Kabeli O., Shmueli Y. (2023). Evolutionary-scale prediction of atomic-level protein structure with a language model. Science.

[36] Khan M.T., Alhartomi M.A. (2024). Digit-Serial DA-Based Fixed-Point RNNs: A Unified Approach for Enhancing Architectural Efficiency. IEEE Trans. Neural Netw. Learn. Syst..

[37] Yalamarthy K.P., Dhall S., Khan M.T., Shaik R.A. (2019). Low-complexity distributed-arithmetic-based pipelined architecture for an LSTM network. IEEE Trans. Very Large Scale Integr. (VLSI) Syst..

[38] Khan M.T., Yantır H.E., Salama K.N., Eltawil A.M. (2022). Architectural trade-off analysis for accelerating LSTM network using Radix-r OBC scheme. IEEE Trans. Circuits Syst. I Regul. Pap..

[39] Alhartomi M.A., Khan M.T., Alzahrani S., Alzahmi A., Shaik R.A., Hazarika J., Alsulami R., Alotaibi A., Al-Harthi M. (2023). Low-area and low-power VLSI architectures for long short-term memory networks. IEEE J. Emerg. Sel. Top. Circuits Syst..

[40] Guo A.B., Akpinaroglu D., Stephens C.A., Grabe M., Smith C.A., Kelly M.J., Kortemme T. (2025). Deep learning–guided design of dynamic proteins. Science.

[41] Jiang Y., Ran X., Yang Z.J. (2023). Data-driven enzyme engineering to identify function-enhancing enzymes. Protein Eng. Des. Sel..

[42] Wang Z., Xie D., Wu D., Luo X., Wang S., Li Y., Yang Y., Li W., Zheng L. (2025). Robust enzyme discovery and engineering with deep learning using CataPro. Nat. Commun..

[43] Wang Y., Cheng L., Zhang Y., Cao Y., Alghazzawi D. (2025). DEKP: A deep learning model for enzyme kinetic parameter prediction based on pretrained models and graph neural networks. Brief. Bioinform..

[44] Li G., Zhang N., Dai X., Fan L. (2024). EnzyACT: A novel deep learning method to predict the impacts of single and multiple mutations on enzyme activity. J. Chem. Inf. Model..

[45] Liu C., Wu J., Chen Y., Liu Y., Zheng Y., Liu L., Zhao J. (2025). Advances in zero-shot prediction-guided enzyme engineering using machine learning. ChemCatChem.

[46] Shi Z., Xu S., Xue S., Chen K., Lu Y., Wang F., Long S., Tian Y., Zhang P., Wang J. (2025). From Machine Learning to Multimodal Models: The AI Revolution in Enzyme Engineering. BioDesign Res..

[47] Farhan M., Hasani I.W., Khafaga D.S.R., Ragab W.M., Ahmed Kazi R.N., Aatif M., Muteeb G., Fahim Y.A. (2025). Enzymes as Catalysts in Industrial Biocatalysis: Advances in Engineering, Applications, and Sustainable Integration. Catalysts.

[48] Xie W.J., Warshel A. (2023). Harnessing generative AI to decode enzyme catalysis and evolution for enhanced engineering. Natl. Sci. Rev..

[49] Mardikoraem M., Wang Z., Pascual N., Woldring D. (2023). Generative models for protein sequence modeling: Recent advances and future directions. Brief. Bioinform..

[50] Barghout R.A., Xu Z., Betala S., Mahadevan R. (2023). Advances in generative modeling methods and datasets to design novel enzymes for renewable chemicals and fuels. Curr. Opin. Biotechnol..

[51] Hawkins-Hooker A., Depardieu F., Baur S., Couairon G., Chen A., Bikard D. (2021). Generating functional protein variants with variational autoencoders. PLoS Comput. Biol..

[52] Schmitt L.T., Paszkowski-Rogacz M., Jug F., Buchholz F. (2022). Prediction of designer-recombinases for DNA editing with generative deep learning. Nat. Commun..

[53] Repecka D., Jauniskis V., Karpus L., Rembeza E., Rokaitis I., Zrimec J., Poviloniene S., Laurynenas A., Viknander S., Abuajwa W. (2021). Expanding functional protein sequence spaces using generative adversarial networks. Nat. Mach. Intell..

[54] Rives A., Meier J., Sercu T., Goyal S., Lin Z., Liu J., Guo D., Ott M., Zitnick C.L., Ma J. (2021). Biological structure and function emerge from scaling unsupervised learning to 250 million protein sequences. Proc. Natl. Acad. Sci. USA.

[55] Munsamy G., Illanes-Vicioso R., Funcillo S., Nakou I.T., Lindner S., Ayres G., Sheehan L.S., Moss S., Eckhard U., Lorenz P. (2024). Conditional language models enable the efficient design of proficient enzymes. bioRxiv.

[56] Wen S., Zheng W., Bornscheuer U.T., Wu S. (2025). Generative artificial intelligence for enzyme design: Recent advances in models and applications. Curr. Opin. Green Sustain. Chem..

[57] Khan M.T., Shaik R.A. (2018). Optimal complexity architectures for pipelined distributed arithmetic-based LMS adaptive filter. IEEE Trans. Circuits Syst. I Regul. Pap..

[58] Khan M.T., Gustafsson O. (2022). ASIC implementation trade-offs for high-speed LMS and block LMS adaptive filters. Proceedings of the 2022 IEEE 65th International Midwest Symposium on Circuits and Systems (MWSCAS).

[59] Grechishnikova D. (2021). Transformer neural network for protein-specific de novo drug generation as a machine translation problem. Sci. Rep..

[60] Dauparas J., Anishchenko I., Bennett N., Bai H., Ragotte R.J., Milles L.F., Wicky B.I., Courbet A., de Haas R.J., Bethel N. (2022). Robust deep learning–based protein sequence design using ProteinMPNN. Science.

[61] Sun H., He L., Deng P., Liu G., Zhao Z., Jiang Y., Cao C., Ju F., Wu L., Liu H. (2025). Accelerating protein engineering with fitness landscape modelling and reinforcement learning. Nat. Mach. Intell..

[62] Pereira T.O., Abbasi M., Arrais J.P. (2023). Enhancing reinforcement learning for de novo molecular design applying self-attention mechanisms. Brief. Bioinform..

[63] Haddad R., Litsa E.E., Liu Z., Yu X., Burkhardt D., Bhisetti G. (2025). Targeted molecular generation with latent reinforcement learning. Sci. Rep..

[64] Koch M., Duigou T., Faulon J.L. (2019). Reinforcement learning for bioretrosynthesis. ACS Synth. Biol..

[65] Damborsky J., Kouba P., Sivic J., Vasina M., Bednar D., Mazurenko S. (2025). Quantum computing for faster enzyme discovery and engineering. Nat. Catal..

[66] Andersson M.P., Jones M.N., Mikkelsen K.V., You F., Mansouri S.S. (2022). Quantum computing for chemical and biomolecular product design. Curr. Opin. Chem. Eng..

[67] Gertig C., Fleitmann L., Hemprich C., Hense J., Bardow A., Leonhard K. (2021). CAT-COSMO-CAMPD: Integrated in silico design of catalysts and processes based on quantum chemistry. Comput. Chem. Eng..

[68] Mandal C., Linthicum D.S. (1993). PROGEN: An automated modelling algorithm for the generation of complete protein structures from the α-carbon atomic coordinates. J. Comput.-Aided Mol. Des..

[69] Krokidis M.G., Koumadorakis D.E., Lazaros K., Ivantsik O., Exarchos T.P., Vrahatis A.G., Kotsiantis S., Vlamos P. (2025). AlphaFold3: An Overview of Applications and Performance Insights. Int. J. Mol. Sci..

[70] Cho Y., Pacesa M., Zhang Z., Correia B.E., Ovchinnikov S. (2025). BoltzDesign1: Inverting All-Atom Structure Prediction Model for Generalized Biomolecular Binder Design. bioRxiv.

[71] Ahern W., Yim J., Tischer D., Salike S., Woodbury S.M., Kim D., Kalvet I., Kipnis Y., Coventry B., Altae-Tran H.R. (2025). Atom Level Enzyme Active Site Scaffolding Using RFdiffusion2. bioRxiv.

[72] Alamdari S., Thakkar N., Van Den Berg R., Tenenholtz N., Strome R., Moses A.M., Lu A.X., Fusi N., Amini A.P., Yang K.K. (2023). Protein Generation with Evolutionary Diffusion: Sequence Is All You Need. bioRxiv.

[73] Yim J., Trippe B.L., De Bortoli V., Mathieu E., Doucet A., Barzilay R., Jaakkola T. (2023). SE(3) Diffusion Model with Application to Protein Backbone Generation. arXiv.

[74] Sánchez B.J., Zhang C., Nilsson A., Lahtvee P.J., Kerkhoven E.J., Nielsen J. (2017). Improving the phenotype predictions of a yeast genome-scale metabolic model by incorporating enzymatic constraints. Mol. Syst. Biol..

[75] Khodayari A., Maranas C.D. (2016). A genome-scale Escherichia coli kinetic metabolic model k-ecoli457 satisfying flux data for multiple mutant strains. Nat. Commun..

[76] Alazmi M. (2024). Enzyme catalytic efficiency prediction: Employing convolutional neural networks and XGBoost. Front. Artif. Intell..

[77] Xuan W., Li X., Gao H., Zhang L., Hu J., Sun L., Kan H. (2025). Artificial intelligence driven platform for rapid catalytic performance assessment of nanozymes. Sci. Rep..

[78] Teng C., Jiang Y., Xu Y., Li Q., Li X., Fan G., Xiong K., Yang R., Zhang C., Ma R. (2019). Improving the thermostability and catalytic efficiency of GH11 xylanase PjxA by adding disulfide bridges. Int. J. Biol. Macromol..

[79] Upadhyay V., Boorla V.S., Maranas C.D. (2023). Rank-ordering of known enzymes as starting points for re-engineering novel substrate activity using a convolutional neural network. Metab. Eng..

[80] Hedstrom L. (2010). Enzyme Specificity and Selectivity. Encyclopedia of Life Sciences (ELS).

[81] Wu L., Qin L., Nie Y., Xu Y., Zhao Y.L. (2022). Computer-aided understanding and engineering of enzymatic selectivity. Biotechnol. Adv..

[82] Arnold F.H. (1993). Engineering proteins for nonnatural environments. FASEB J..

[83] Bloom J.D., Arnold F.H. (2009). In the light of directed evolution: Pathways of adaptive protein evolution. Proc. Natl. Acad. Sci. USA.

[84] Song Z., Zhang Q., Wu W., Pu Z., Yu H. (2023). Rational design of enzyme activity and enantioselectivity. Front. Bioeng. Biotechnol..

[85] Varadarajan N., Gam J., Olsen M.J., Georgiou G., Iverson B.L. (2005). Engineering of protease variants exhibiting high catalytic activity and exquisite substrate selectivity. Proc. Natl. Acad. Sci. USA.

[86] Kim S., Ga S., Bae H., Sluyter R., Konstantinov K., Shrestha L.K., Kim Y.H., Kim J.H., Ariga K. (2024). Multidisciplinary approaches for enzyme biocatalysis in pharmaceuticals: Protein engineering, computational biology, and nanoarchitectonics. EES Catal..

[87] Li T., Liang J., Ambrogelly A., Brennan T., Gloor G., Huisman G., Lalonde J., Lekhal A., Mijts B., Muley S. (2012). Efficient, chemoenzymatic process for manufacture of the boceprevir bicyclic [3.1.0] proline intermediate based on amine oxidase-catalyzed desymmetrization. J. Am. Chem. Soc..

[88] Wilson C., Agard D.A. (1991). Engineering substrate specificity. Curr. Opin. Struct. Biol..

[89] Carter P., Wells J.A. (1987). Engineering enzyme specificity by “substrate-assisted catalysis”. Science.

[90] Chakravorty D., Khan M.F., Patra S. (2017). Multifactorial level of extremostability of proteins: Can they be exploited for protein engineering?. Extremophiles.

[91] Khan M.F. (2025). Enhancing stability of enzymes for industrial applications: Molecular insights and emerging approaches. World J. Microbiol. Biotechnol..

[92] Khan M.F., Patra S. (2018). Deciphering the rationale behind specific codon usage pattern in extremophiles. Sci. Rep..

[93] Saha P., Khan M.F., Patra S. (2018). Truncated α-amylase: An improved candidate for textile processing. Prep. Biochem. Biotechnol..

[94] Chakravorty D., Khan M.F., Patra S. (2017). Thermostability of proteins revisited through machine learning methodologies: From nucleotide sequence to structure. Curr. Biotechnol..

[95] Kang L., Wu B., Zhou B., Tan P., Kang Y.K., Yan Y., Zong Y., Li S., Liu Z., Hong L. (2025). AI-enabled alkaline-resistant evolution of protein to apply in mass production. eLife.

[96] Bian J., Tan P., Nie T., Hong L., Yang G.Y. (2024). Optimizing enzyme thermostability by combining multiple mutations using protein language model. MLife.

[97] Muellers S.N., Allen K.N., Whitty A. (2023). MEnTaT: A machine-learning approach for the identification of mutations to increase protein stability. Proc. Natl. Acad. Sci. USA.

[98] Zheng N., Cai Y., Zhang Z., Zhou H., Deng Y., Du S., Tu M., Fang W., Xia X. (2025). Tailoring industrial enzymes for thermostability and activity evolution by the machine learning-based iCASE strategy. Nat. Commun..

[99] Dou Z., Sun Y., Jiang X., Wu X., Li Y., Gong B., Wang L. (2023). Data-driven strategies for the computational design of enzyme thermal stability: Trends, perspectives, and prospects. Acta Biochim. Biophys. Sin..

[100] Teng R., Zhang J., Tu Z., He Q., Li Y. (2024). Computer-Aided Design to Improve the Thermal Stability of *Rhizomucor miehei* Lipase. Foods.

[101] Son H., Seo H., Han S., Kim S.M., Pham L.T.M., Khan M.F., Sung H.J., Kang S.H., Kim K.J., Kim Y.H. (2021). Extra disulfide and ionic salt bridge improves the thermostability of lignin peroxidase H8 under acidic condition. Enzyme Microb. Technol..

[102] Wang L., Watermeyer J.M., Mulelu A.E., Sewell B.T., Benedik M.J. (2012). Engineering pH-tolerant mutants of a cyanide dihydratase. Appl. Microbiol. Biotechnol..

[103] Crum M.A., Sewell B.T., Benedik M.J. (2016). Bacillus pumilus cyanide dihydratase mutants with higher catalytic activity. Front. Microbiol..

[104] Idicula-Thomas S., Balaji P.V. (2005). Understanding the relationship between the primary structure of proteins and its propensity to be soluble on overexpression in *Escherichia coli*. Protein Sci..

[105] Chen J.P., Gong J.S., Su C., Li H., Xu Z.H., Shi J.S. (2023). Improving the soluble expression of difficult-to-express proteins in prokaryotic expression system via protein engineering and synthetic biology strategies. Metab. Eng..

[106] Jo B.H. (2024). Improved solubility and stability of a thermostable carbonic anhydrase via fusion with marine-derived intrinsically disordered solubility enhancers. Int. J. Mol. Sci..

[107] Han X., Wang X., Zhou K. (2019). Develop machine learning-based regression predictive models for engineering protein solubility. Bioinformatics.

[108] Hirose S., Noguchi T. (2013). ESPRESSO: A system for estimating protein expression and solubility in protein expression systems. Proteomics.

[109] Hebditch M., Carballo-Amador M.A., Charonis S., Curtis R., Warwicker J. (2017). Protein–Sol: A web tool for predicting protein solubility from sequence. Bioinformatics.

[110] Khurana S., Rawi R., Kunji K., Chuang G.Y., Bensmail H., Mall R. (2018). DeepSol: A deep learning framework for sequence-based protein solubility prediction. Bioinformatics.

[111] Klesmith J.R., Bacik J.P., Wrenbeck E.E., Michalczyk R., Whitehead T.A. (2017). Trade-offs between enzyme fitness and solubility illuminated by deep mutational scanning. Proc. Natl. Acad. Sci. USA.

[112] Khan M.F., Kundu D., Hazra C., Patra S. (2019). A strategic approach of enzyme engineering by attribute ranking and enzyme immobilization on zinc oxide nanoparticles to attain thermostability in mesophilic Bacillus subtilis lipase for detergent formulation. Int. J. Biol. Macromol..

[113] Scherer M., Fleishman S.J., Jones P.R., Dandekar T., Bencurova E. (2021). Computational enzyme engineering pipelines for optimized production of renewable chemicals. Front. Bioeng. Biotechnol..

[114] Zanghellini A. (2014). De novo computational enzyme design. Curr. Opin. Biotechnol..

[115] Cui X.C., Zheng Y., Liu Y., Yuchi Z., Yuan Y.J. (2025). AI-driven de novo enzyme design: Strategies, applications, and future prospects. Biotechnol. Adv..

[116] Tiwari V. (2016). In vitro engineering of novel bioactivity in natural enzymes. Front. Chem..

[117] Hua C., Lu J., Liu Y., Zhang O., Tang J., Ying R., Jin W., Wolf G., Precup D., Zheng S. (2024). Reaction-conditioned de novo enzyme design with Genzyme. arXiv.

[118] Braun M., Tripp A., Chakatok M., Kaltenbrunner S., Fischer C., Stoll D., Bijelic A., Elaily W., Totaro M.G., Moser M. (2025). Computational enzyme design by catalytic motif scaffolding. Nature.

[119] Ruffolo J.A., Nayfach S., Gallagher J., Bhatnagar A., Beazer J., Hussain R., Russ J., Yip J., Hill E., Pacesa M. (2025). Design of Highly Functional Genome Editors by Modelling CRISPR-Cas Sequences. Nature.

[120] Nguyen E., Poli M., Durrant M.G., Kang B., Katrekar D., Li D.B., Bartie L.J., Thomas A.W., King S.H., Brixi G. (2024). Sequence Modeling and Design from Molecular to Genome Scale with Evo. Science.

[121] Kurihara K., Umezawa K., Donnelly A.E., Sperling B., Liao G., Hecht M.H., Arai R. (2023). Crystal structure and activity of a de novo enzyme, ferric enterobactin esterase Syn-F4. Proc. Natl. Acad. Sci. USA.

[122] Orsi E., von Borzyskowski L.S., Noack S., Nikel P.I., Lindner S.N. (2024). Automated in vivo enzyme engineering accelerates biocatalyst optimization. Nat. Commun..

[123] Wang X., Xu K., Tan Y., Liu S., Zhou J. (2023). Possibilities of using de novo design for generating diverse functional food enzymes. Int. J. Mol. Sci..

[124] Hossack E.J., Hardy F.J., Green A.P. (2023). Building enzymes through design and evolution. ACS Catal..

[125] Naddaf M. (2025). Scientists Use AI to Design Life-Like Enzymes from Scratch. Nature.

[126] Ming Y., Wang W., Yin R., Zeng M., Tang L., Tang S., Li M. (2023). A review of enzyme design in catalytic stability by artificial intelligence. Brief. Bioinform..

[127] Siedentop R., Siska M., Möller N., Lanzrath H., von Lieres E., Lütz S., Rosenthal K. (2023). Bayesian optimization for an ATP-regenerating in vitro enzyme cascade. Catalysts.

[128] Putz S., Teetz N., Abt M., Jerono P., Meurer T., Franzreb M. (2025). Optimized machine learning for autonomous enzymatic reaction intensification in a self-driving lab. Biotechnol. Bioeng..

[129] Gantz M., Neun S., Medcalf E.J., van Vliet L.D., Hollfelder F. (2023). Ultrahigh-throughput enzyme engineering and discovery in in vitro compartments. Chem. Rev..

[130] Savile C.K., Janey J.M., Mundorff E.C., Moore J.C., Tam S., Jarvis W.R., Colbeck J.C., Krebber A., Fleitz F.J., Brands J. (2010). Biocatalytic asymmetric synthesis of chiral amines from ketones applied to sitagliptin manufacture. Science.

[131] Desai A.A. (2011). Sitagliptin manufacture: A compelling tale of green chemistry, process intensification, and industrial asymmetric catalysis. Angew. Chem. Int. Ed..

[132] Nielsen J., Keasling J.D. (2016). Engineering cellular metabolism. Cell.

[133] Dusséaux S., Wajn W.T., Liu Y., Ignea C., Kampranis S.C. (2020). Transforming yeast peroxisomes into microfactories for the efficient production of high-value isoprenoids. Proc. Natl. Acad. Sci. USA.

[134] Guo Q., Yang Y.X., Li D.X., Ji X.J., Wu N., Wang Y.T., Ye C., Shi T.Q. (2024). Advances in multi-enzyme co-localization strategies for the construction of microbial cell factory. Biotechnol. Adv..

[135] Delebecque C.J., Lindner A.B., Silver P.A., Aldaye F.A. (2011). Organization of intracellular reactions with rationally designed RNA assemblies. Science.

[136] Boob A.G., Chen J., Zhao H. (2024). Enabling pathway design by multiplex experimentation and machine learning. Metab. Eng..

[137] Lee M.E., Aswani A., Han A.S., Tomlin C.J., Dueber J.E. (2013). Expression-level optimization of a multi-enzyme pathway in the absence of a high-throughput assay. Nucleic Acids Res..

[138] Xu W., Cai J., Wu W., Yuan Q., Mao Z., Ma H. (2025). Improving metabolic engineering design with enzyme-thermo optimization. Metab. Eng..

[139] Gao D.Y., Sun X.B., Liu M.Q., Liu Y.N., Zhang H.E., Shi X.L., Li Y.N., Wang J.K., Yin S.J., Wang Q. (2019). Characterization of thermostable and chimeric enzymes via isopeptide bond-mediated molecular cyclization. J. Agric. Food Chem..

[140] Radley E., Davidson J., Foster J., Obexer R., Bell E.L., Green A.P. (2023). Engineering enzymes for environmental sustainability. Angew. Chem. Int. Ed..

[141] Khan M.F. (2025). Recent progress and challenges in microbial defluorination and degradation for sustainable remediation of fluorinated xenobiotics. Processes.

[142] Khan M.F., Rama M., Murphy C.D. (2025). Biodegradation of fluorinated β-triketone herbicide tembotrione by a bacterial–fungal consortium. Biocatal. Agric. Biotechnol..

[143] Khan M.F., Hof C., Niemcová P., Murphy C.D. (2023). Recent advances in fungal xenobiotic metabolism: Enzymes and applications. World J. Microbiol. Biotechnol..

[144] Khan M.F., Murphy C.D. (2022). Nitroreduction of flutamide by *Cunninghamella elegans* NADPH: Cytochrome P450 reductase. Biochem. Biophys. Rep..

[145] Khan M.F. (2025). Recent advances in microbial enzyme applications for sustainable textile processing and waste management. Sci.

[146] Griffiths A.D., Tawfik D.S. (2003). Directed evolution of an extremely fast phosphotriesterase by in vitro compartmentalization. EMBO J..

[147] Floor R.J., Wijma H.J., Colpa D.I., Ramos---Silva A., Jekel P.A., Szymański W., Feringa B.L., Marrink S.J., Janssen D.B. (2014). Computational library design for increasing haloalkane dehalogenase stability. ChemBioChem.

[148] Khan M.F. (2025). Fungi for sustainable pharmaceutical remediation: Enzymatic innovations, challenges, and applications—A review. Processes.

[149] Jacquet P., Hiblot J., Daudé D., Bergonzi C., Gotthard G., Armstrong N., Chabrière E., Elias M. (2017). Rational engineering of a native hyperthermostable lactonase into a broad spectrum phosphotriesterase. Sci. Rep..

[150] Poirier L., Pinault L., Armstrong N., Ghigo E., Daudé D., Chabrière E. (2019). Evaluation of a robust engineered enzyme towards organophosphorus insecticide bioremediation using planarians as biosensors. Chem.-Biol. Interact..

[151] Sung H.J., Khan M.F., Kim Y.H. (2019). Recombinant lignin peroxidase-catalyzed decolorization of melanin using in situ generated H2O2 for application in whitening cosmetics. Int. J. Biol. Macromol..

[152] Ndochinwa O.G., Wang Q.Y., Amadi O.C., Nwagu T.N., Nnamchi C.I., Okeke E.S., Moneke A.N. (2024). Current status and emerging frontiers in enzyme engineering: An industrial perspective. Heliyon.

[153] Victorino da Silva Amatto I., Gonsales da Rosa-Garzon N., Antônio de Oliveira Simões F., Santiago F., Pereira da Silva Leite N., Raspante Martins J., Cabral H. (2022). Enzyme engineering and its industrial applications. Biotechnol. Appl. Biochem..

[154] Alcalde M. (2017). Directed Enzyme Evolution: Advances and Applications.

[155] Shelat N.Y., Parhi S., Ostermeier M. (2017). Development of a cancer-marker activated enzymatic switch from the herpes simplex virus thymidine kinase. Protein Eng. Des. Sel..

[156] Khan M.F., Murphy C.D. (2021). Bacterial degradation of the anti-depressant drug fluoxetine produces trifluoroacetic acid and fluoride ion. Appl. Microbiol. Biotechnol..

[157] Shahbazmohammadi H., Sardari S., Lari A., Omidinia E. (2019). Engineering an efficient mutant of Eupenicillium terrenum fructosyl peptide oxidase for the specific determination of hemoglobin A1c. Appl. Microbiol. Biotechnol..

[158] Giessel A., Dousis A., Ravichandran K., Smith K., Sur S., McFadyen I., Zheng W., Licht S. (2022). Therapeutic Enzyme Engineering Using a Generative Neural Network. Sci. Rep..

[159] Huisman G.W., Collier S.J. (2013). On the development of new biocatalytic processes for practical pharmaceutical synthesis. Curr. Opin. Chem. Biol..

[160] Huisman G.W., Liang J., Krebber A. (2010). Practical chiral alcohol manufacture using ketoreductases. Curr. Opin. Chem. Biol..

[161] Midelfort K.S., Kumar R., Han S., Karmilowicz M.J., McConnell K., Gehlhaar D.K., Mistry A., Chang J.S., Anderson M., Villalobos A. (2013). Redesigning and characterizing the substrate specificity and activity of Vibrio fluvialis aminotransferase for the synthesis of imagabalin. Protein Eng. Des. Sel..

[162] Ghislieri D., Green A.P., Pontini M., Willies S.C., Rowles I., Frank A., Grogan G., Turner N.J. (2013). Engineering an enantioselective amine oxidase for the synthesis of pharmaceutical building blocks and alkaloid natural products. J. Am. Chem. Soc..

[163] Fenel F., Leisola M., Jänis J., Turunen O. (2004). A de novo designed N-terminal disulphide bridge stabilizes the Trichoderma reesei endo-1,4-β-xylanase II. J. Biotechnol..

[164] Han N., Ma Y., Mu Y., Tang X., Li J., Huang Z. (2019). Enhancing thermal tolerance of a fungal GH11 xylanase guided by B-factor analysis and multiple sequence alignment. Enzyme Microb. Technol..

[165] Zhou C., Xue Y., Ma Y. (2015). Evaluation and directed evolution for thermostability improvement of a GH13 thermo-stable α-glucosidase from Thermus thermophilus TC11. BMC Biotechnol..

[166] Lin L.L., Liu J.S., Wang W.C., Chen S.H., Huang C.C., Lo H.F. (2008). Glutamic acid 219 is critical for the thermostability of a truncated α-amylase from alkaliphilic and thermophilic Bacillus sp. strain TS-23. World J. Microbiol. Biotechnol..

[167] Duan X., Chen J., Wu J. (2013). Improving the thermostability and catalytic efficiency of Bacillus deramificans pullulanase by site-directed mutagenesis. Appl. Environ. Microbiol..

[168] Yu X.J., Huang C.Y., Xu X.D., Chen H., Liang M.J., Xu Z.X., Xu H.X., Wang Z. (2020). Protein engineering of a pyridoxal-5′-phosphate-dependent l-aspartate-α-decarboxylase from *Tribolium castaneum* for β-alanine production. Molecules.

[169] Lee S.C., Chang Y., Shin D.M., Han J., Seo M.H., Fazelinia H., Maranas C.D., Kim H.S. (2009). Designing the substrate specificity of D-hydantoinase using a rational approach. Enzyme Microb. Technol..

[170] Hegazy U.M., El-Khonezy M.I., Shokeer A., Abdel-Ghany S.S., Bassuny R.I., Barakat A.Z., Salama W.H., Azouz R.A., Fahmy A.S. (2019). Revealing of a novel xylose-binding site of *Geobacillus stearothermophilus* xylanase by directed evolution. J. Biochem..

[171] Madan B., Mishra P. (2014). Directed evolution of Bacillus licheniformis lipase for improvement of thermostability. Biochem. Eng. J..

[172] Zhao H.Y., Feng H. (2018). Engineering Bacillus pumilus alkaline serine protease to increase its low-temperature proteolytic activity by directed evolution. BMC Biotechnol..

[173] Jakob F., Martinez R., Mandawe J., Hellmuth H., Siegert P., Maurer K.H., Schwaneberg U. (2013). Surface charge engineering of a Bacillus gibsonii subtilisin protease. Appl. Microbiol. Biotechnol..

[174] Dror A., Shemesh E., Dayan N., Fishman A. (2014). Protein engineering by random mutagenesis and structure-guided consensus of *Geobacillus stearothermophilus* lipase T6 for enhanced stability in methanol. Appl. Environ. Microbiol..

[175] Zhao G., Wang J., Tang Q., Lan D., Wang Y. (2018). Improving the catalytic activity and thermostability of MAS1 lipase by alanine substitution. Mol. Biotechnol..

[176] Ma C., Wu R., Huang R., Jiang W., You C., Zhu L., Zhu Z. (2019). Directed evolution of a 6-phosphogluconate dehydrogenase for operating an enzymatic fuel cell at lowered anodic pHs. J. Electroanal. Chem..

[177] Ali M., Husain Q., Alam N., Ahmad M. (2018). Nano-peroxidase fabrication on cation exchanger nanocomposite: Augmenting catalytic efficiency and stability for the decolorization and detoxification of Methyl Violet 6B dye. Sep. Purif. Technol..

[178] Harford-Cross C.F., Carmichael A.B., Allan F.K., England P.A., Rouch D.A., Wong L.L. (2000). Protein engineering of cytochrome P450cam (CYP101) for the oxidation of polycyclic aromatic hydrocarbons. Protein Eng..

[179] Syed K., Porollo A., Miller D., Yadav J.S. (2013). Rational engineering of the fungal P450 monooxygenase CYP5136A3 to improve its oxidizing activity toward polycyclic aromatic hydrocarbons. Protein Eng. Des. Sel..

[180] Bilal M., Iqbal H.M. (2020). State-of-the-art strategies and applied perspectives of enzyme biocatalysis in food sector—current status and future trends. Crit. Rev. Food Sci. Nutr..

[181] Vojcic L., Pitzler C., Körfer G., Jakob F., Martinez R., Maurer K.H., Schwaneberg U. (2015). Advances in protease engineering for laundry detergents. New Biotechnol..

[182] Khan M.F., Murphy C.D. (2023). Environmental remediation by novel nanomaterials and fungi with high-degradation capacity of hazardous contaminants. Bio and Nanoremediation of Hazardous Environmental Pollutants.

[183] Khan M.F., Liao J., Liu Z., Chugh G. (2025). Bacterial cytochrome P450 involvement in the biodegradation of fluorinated pyrethroids. J. Xenobiot..

[184] Agrawal K., Bhatt A., Chaturvedi V., Verma P. (2020). Bioremediation: An effective technology toward a sustainable environment via the remediation of emerging environmental pollutants. Emerging Technologies in Environmental Bioremediation.

[185] Singh N., Malik S., Gupta A., Srivastava K.R. (2021). Revolutionizing enzyme engineering through artificial intelligence and machine learning. Emerg. Top. Life Sci..

[186] Sapoval N., Aghazadeh A., Nute M.G., Antunes D.A., Balaji A., Baraniuk R., Barberan C., Dannenfelser R., Dun C., Edrisi M. (2022). Current progress and open challenges for applying deep learning across the biosciences. Nat. Commun..

[187] Nugroho H. (2023). A review: Data quality problem in predictive analytics. IJAIT (Int. J. Appl. Inf. Technol.).

[188] Heid E., Probst D., Green W.H., Madsen G.K. (2023). EnzymeMap: Curation, validation and data-driven prediction of enzymatic reactions. Chem. Sci..

[189] Probst D., Manica M., Nana Teukam Y.G., Castrogiovanni A., Paratore F., Laino T. (2022). Biocatalysed synthesis planning using data-driven learning. Nat. Commun..

[190] Tipton K.F., Armstrong R.N., Bakker B.M., Bairoch A., Cornish-Bowden A., Halling P.J., Hofmeyr J.H., Leyh T.S., Kettner C., Raushel F.M. (2014). Standards for Reporting Enzyme Data: The STRENDA Consortium: What it aims to do and why it should be helpful. Perspect. Sci..

[191] Wilkinson M.D., Dumontier M., Aalbersberg I.J., Appleton G., Axton M., Baak A., Blomberg N., Boiten J.W., da Silva Santos L.B., Bourne P.E. (2016). The FAIR Guiding Principles for scientific data management and stewardship. Sci. Data.

[192] Sandve G.K., Nekrutenko A., Taylor J., Hovig E. (2013). Ten simple rules for reproducible computational research. PLoS Comput. Biol..

[193] Matsuta Y., Ito M., Tohsato Y. (2013). ECOH: An enzyme commission number predictor using mutual information and a support vector machine. Bioinformatics.

[194] Baylon J.L., Cilfone N.A., Gulcher J.R., Chittenden T.W. (2019). Enhancing retrosynthetic reaction prediction with deep learning using multiscale reaction classification. J. Chem. Inf. Model..

[195] Tavakoli M., Shmakov A., Ceccarelli F., Baldi P. (2022). Rxn hypergraph: A hypergraph attention model for chemical reaction representation. arXiv.

[196] Zeng K., Liu X., Zhang Y., Yang X., Jin Y., Xu Y. (2024). Learning chemical reaction representation with reactant-product alignment. arXiv.

[197] Copley S.D. (2003). Enzymes with extra talents: Moonlighting functions and catalytic promiscuity. Curr. Opin. Chem. Biol..

[198] Delépine B., Duigou T., Carbonell P., Faulon J.L. (2018). RetroPath2.0: A retrosynthesis workflow for metabolic engineers. Metab. Eng..

[199] Dönertaş H.M., Martínez Cuesta S., Rahman S.A., Thornton J.M. (2016). Characterising complex enzyme reaction data. PLoS ONE.

[200] Schnoes A.M., Brown S.D., Dodevski I., Babbitt P.C. (2009). Annotation error in public databases: Misannotation of molecular function in enzyme superfamilies. PLoS Comput. Biol..

[201] Kulmanov M., Hoehndorf R. (2020). DeepGOPlus: Improved protein function prediction from sequence. Bioinformatics.

[202] Heijnen J.J., Verheijen P.J. (2013). Parameter identification of in vivo kinetic models: Limitations and challenges. Biotechnol. J..

